# Novel secretome-to-transcriptome integrated or secreto-transcriptomic approach to reveal liquid biopsy biomarkers for predicting individualized prognosis of breast cancer patients

**DOI:** 10.1186/s12920-019-0530-7

**Published:** 2019-05-30

**Authors:** J. Astor Ankney, Ling Xie, John A. Wrobel, Li Wang, Xian Chen

**Affiliations:** 1 Department of Biochemistry & Biophysics, University of North Carolina at Chapel Hill, Chapel Hill, NC 27599 USA; 2 Lineberger Comprehensive Cancer Center, University of North Carolina at Chapel Hill, Chapel Hill, NC 27599 USA

**Keywords:** Label-free quantitative proteomics, Protein secretion, Multi-omics correlations, Secretion-correlated expression pattern, TCGA, Patient survival analysis

## Abstract

**Background:**

Presently, a 50-gene expression model (PAM50) serves as a breast cancer (BC) subtype classifier that is insufficient to distinguish, within each single PAM50-classified subtype, patient subpopulations having different prognosis. There is a pressing need for inexpensive and minimally invasive biomarker tests to easily and accurately predict individuals’ clinical outcomes and response to treatments. Although quantitative proteomic approaches have been developed to identify/profile proteins secreted (secretome) from various cancer cell lines in vitro, missing are the clinicopathological relevance and the associated prognostic value of these secretomic identifications.

**Methods:**

To discover biomarkers to predict individualized prognosis we introduce a new multi-omics (secreto-transcriptomics) method that identifies, in their oncogenically secreted states, candidate markers of BC subtypes whose genes bear patient-specific mRNA expression alterations of prognostic significance. First, we used label-free quantitative (LFQ) proteomics to identify the proteins showing BC-subtypic secretion from a series of BC cell lines representing major BC-subtypes. To determine and externally validate the prognostic value of these secreted proteins, we developed a secreto-transcriptomic approach that discovered a PAM50-subtypic Secretion-Correlated mRNA Expression Pattern (SeCEP) wherein the PAM50-subtypic secretion of select proteins statistically correlated with *cis*-mRNA expression of their encoding genes in patients of the corresponding PAM50-subtypes. Kaplan-Meier analysis of SeCEP genes was used to identify new liquid biopsy biomarkers for predicting individualized prognosis.

**Results:**

The mRNA expression-to-secretion correlation (SeCEP) pinpointed multiple genes that are fully translated into the oncogenically active secretome in a PAM50-subtypic manner. Further, multiple SeCEP genes in distinct combinations or panels of multiple SeCEP genes were identified as ‘systems prognostic markers’ that showed mRNA co-overexpression patterns in the distinct subpopulations of PAM50-subtypic patients with poor prognosis or high-risk of relapse. Thus, our secreto-transcriptomic approach statistically linked BC subtypic secretome genes with patient-specific information about their mRNA expression alterations and significantly improved the sensitivity and specificity in patient stratification in the context of clinical outcomes or prognosis.

**Conclusions:**

By combining LFQ secretome screening with proteo-transcriptomic retrospective analysis of patient data our integrated multi-omics approach bypasses costly, tedious, genome-wide fishing and predictive modeling that are commonly required to distinguish a few prognostically altered genes from thousands of other non-BC related genes in a genome.

**Electronic supplementary material:**

The online version of this article (10.1186/s12920-019-0530-7) contains supplementary material, which is available to authorized users.

## Background

Breast cancer (BC) is the most prevalent type of cancer among women in the United States, with over 200,000 new diagnoses of invasive BC per year. [1] However, significant heterogeneity among BC tumors contributes to highly variable clinical pathology and patient outcomes, ultimately confounding efforts toward precision diagnosis and prognosis. [2] A 50-gene expression pattern has been used to classify five molecular subtypes or PAM50-subtypes, including basal-like/triple-negative (BLBC/TNBC), luminal-A and -B, Her2+, and normal-like BC. [3] Within these molecular subtypes, the luminal subtype accounts for approximately half of all tumors, [4] and BLBC/TNBC is the most aggressive form of the disease with the overall worst survival rate. [5] However, these gene-expression signatures are inadequate to resolve interpatient heterogeneity, and patient subpopulations with different clinical outcomes cannot be stratified within each BC or PAM50 subtype. [6] These limitations arise because disease onset is directly governed by phenotype-specific proteomic changes [7, 8] which cannot be measured using genomic/transcriptomic tools or data alone. Because prognoses of BC patients cannot be easily discerned, there is an urgent need for individualized/personalized biomarkers that predict patient-specific survival rates and therapeutic response so that standard chemotherapy may be replaced by more effective and precise treatment. Because tumor cells secrete and shed characteristic proteins at a higher rate than healthy cells, and many of these proteins enter circulation to play extracellular regulatory roles, [9, 10] proteins secreted or shed by cancer cells (the “cancer secretome”), could be phenotypic biomarkers. More importantly, in clinical practice these tumor-characteristically secreted proteins may be detectable in blood or other bodily fluids in a non-invasive manner. [10]

Secreted proteins, which constitute approximately 10% of the human proteome, play an important role in normal physiological processes including cell signaling, immune defense, and blood coagulation. [11] Further, when deregulated, secreted proteins are critical participants in pathological processes such as cancer angiogenesis, invasion, and metastasis. [11] Also, secreted factors have been increasingly recognized for their role in the mechanisms of drug response. [12] The studies of BC secretomes using nanoliter liquid chromatograph tandem mass spectrometry (nanoLC-MS/MS) to sample conditioned medium from cell lines, tumor/tissue interstitial fluid, or tumor proximal body fluids have been reported. [9, 13–16] However, few proteins identified in these BC secretomes have established correlation with patient-specific clinical outcomes.

Technically, at the current level of sensitivity, mass spectrometry can detect only very limited regions of each individual protein, further limiting information about patient-specific alterations in these secreted proteins. [17, 18] Therefore, due to either the low phenotypic accuracy of genomics/transcriptomics or the low phenotypic coverage of proteomics, approaches that employ only single-omics methods will necessarily fail to identify biomarkers of patient-specific alterations which distinguish patient subpopulations having different clinical outcomes or prognoses. To overcome these single-omics limitations for identifying new biomarkers to predict individualized prognosis in non-invasive, blood-based tests, we developed a new multi-omics method, termed *secreto-transcriptomics*, to identify the BC-subtypic secreted proteins that are encoded by genes bearing patient-specific mRNA expression patterns of prognostic significance.

Strategically in advance, our secreto-transcriptomic approach bypasses both the inability of conventional MS to connect genotype to phenotype and the inability of MS to fully identify patient-specific proteomic alterations, integrating oncogenic (tumorigenic) multi-omics data for efficient de novo discovery of personalized/individualized prognostic markers. In the clinic, these markers may be used to stratify patients, within single PAM50 subtypes, into different prognostic groups, and predict treatment benefit and/or outcome with patient-specific or individualized sensitivity and specificity before any therapeutic decision for newly diagnosed breast cancer.

## Methods

### Chemicals and reagents

Cell culture media and fetal bovine serum were obtained from Gibco. All other components of cell culture media and protease inhibitor cocktails were purchased from Sigma (St, Louis, MO). Trypsin was purchased from Promega. All chemicals were HPLC-grade unless specifically indicated. All cell lines including MCF10A, MCF7, MDA-231, T47D, and HCC1806 were purchased from ATCC (Manassas,VA).

### Secreted protein collection from cell lines

MCF10A cells were cultured in DMEM/F12 supplemented with 5% horse serum, 20 ng/mL epidermal growth factor, 50 ng/mL cholera toxin, 500 ng/mL hydrocortisone, and 2 μg/mL insulin. MCF7 and MDA-231 cells were cultured in DMEM containing 10% fetal bovine serum. T47D and HCC1806 cells were maintained in RPMI supplemented with 10% fetal bovine serum. When cells reached approximately 70% confluence, the growth media was removed, cells were washed twice with PBS, and serum-free media without phenol red was added to the plate. After 24 h the conditioned media were collected and centrifuged at 500 x g for 5 min to remove cellular debris, then the supernatant was syringe-filtered with 0.2 μm 13 mm diameter polytetrafluoroethylene filters (VWR International) and transferred to fresh tubes. Samples were stored at − 80 °C until further processed. After thawing, proteins were concentrated by trichloroacetic acid/sodium deoxycholate precipitation. Briefly, 1/10 of the sample volume of 0.15% sodium deoxycholate was added to each sample, then tubes were incubated on ice for 15 min. Next, 1/10 of the original sample volume of cold 72% trichloroacetic acid was added and the tubes were incubated on ice for 15 min. Samples were centrifuged for 10 min at max speed, 4 °C. The pellets were washed in cold acetone and air dried until no residual odor was detected. Next, the pellets were resuspended in 50 μl buffer (8 M Urea, 50 mM Tris-HCl pH 8.0, 150 mM NaCl), reduced with dithiothreitol (5 mM final) for 30 min at room temperature, and alkylated with iodoacetamide (15 mM final) for 45 min in the dark at room temperature. Alkylation was quenched with dithiothreitol (10 mM final). Samples were diluted 4-fold with 25 mM Tris-HCl pH 8.0, 1 mM CaCl_2_ and digested with 500 ng trypsin overnight at room temperature. Peptides were desalted on a StageTip containing a 4 × 1 mm C18 extraction disk (3 M) and dried. [19]

### LC-MS/MS analysis

LC-MS/MS analysis was performed as previously described. [20] Briefly, desalted peptides were dissolved in 20 μl 0.1% formic acid (Thermo-Fisher). An injection of 2 μl was analyzed by an Easy nanoLC 1000 with a 15 cm C18 reverse phase column (15 cm × 75 μm ID, C18, 2 μm, Acclaim Pepmap RSLC, Thermo-Fisher) coupled to a Q-Exactive Orbitrap mass spectrometer (Thermo Fisher Scientific, San Jose, CA). Peptides were eluted at a constant flow rate of 300 nl/min with a gradient of 2–30% buffer B (acetonitrile and 0.1% formic acid) for 30 min, 30–80% buffer B for 5 min, and 80% B for 10 min. Experiments were performed using a data-dependent top 20 method in positive-ion mode. Full MS was performed at a resolution of 70,000 and m/z = 200. Up to the top 20 most intense ions with charge ≥2 from full MS were selected with an isolation window of 2.0 m/z and higher energy collisional dissociation was used to fragment peptides at a normalized collision energy of 27 eV. The maximum ion injection time for full MS was 250 ms with ion target value of 1e6, and maximum ion injection time for MS/MS was 120 ms with ion target value of 2e5. Selected sequenced ions were dynamically excluded for 20 s.

### Mass spec data and LFQ analysis

Mass spectral processing and peptide identification were performed on the Andromeda search engine in MaxQuant software (Version 1.5.3.17) against a human UniProt database. Cysteine carbamidomethylation was set as a defined modification, and methionine oxidation and protein amino-terminal acetylation were set as dynamic modifications. Peptide inference was made with a false discovery rate (FDR) of 1% and peptides were assigned to proteins with FDR of 5%. At least 7 amino acids were required with no more than two missed cleavages. The precursor ion mass tolerance was 8 ppm and the fragment ion mass tolerance was 0.5 Da. Experiments were conducted in multiple replicates (three biological replicates each with two technical replicates) using a match between runs option enabled and time window at 0.7 min. Data processing and statistical analysis were performed on Perseus (Version 1.5.1.6). [21]

### Analysis of functional category and networks of subtype-specific secreted proteins

The biological processes and molecular functions of secretome proteins were categorized by Ingenuity Pathway Analysis (IPA) [22] and STRING [23] similar to previously described. [24]

### TCGA and METABRIC data sets

TCGA and METABRIC data were retrieved from cBioPortal [25, 26] using the ‘cgdsr’ R package (version 1.2.6). [27] Complete samples (case list id = brca_tcga_pub2015_3way_complete / brca_tcga_pub2015_freeze) with mutation, copy-number, and mRNA expression data provided (*N* = 816) from the TCGA cancer study brca_tcga_pub2015 (Breast Invasive Carcinoma) were used. [28] The mRNA expression data sets for secretome genes were obtained from TCGA Genetic Profile: brca_tcga_pub2015_rna_seq_v2_mrna_median_Zscores, containing mRNA expression Z-scores compared with diploid tumors (diploid for each gene). Clinical data including information on estrogen receptor (ER) status, progesterone receptor (PR) status, HER2 enrichment (HER2) status, and patient survival were obtained from TCGA case list id: brca_tcga_pub2015_3way_complete / brca_tcga_pub2015_freeze. The PAM50 subtype assigned to each patient and other additional clinical datawere obtained from “Additional file 9: Table S1” in the TCGA publication. [28] METABRIC [29, 30] data was acquired using case list id = brca_metabric_cnaseq (samples with mRNA, GISTIC, mutational data), gene profile = brca_metabric_mrna_U133_Zscores (for mRNA expression). Of the 2051 samples in the METABRIC case list, 1866 for which there was survival data were used in the analysis.

### Heatmap construction

The ‘ComplexHeatmap’ R package (version 1.12.0) was used to construct the heatmap of SeCEP gene mRNA expression levels from the TCGA BRCA datasets. [31] The euclidean distance method and Ward clustering method (option ward. D2 in R’s hclust function) were used for hierarchical clustering.

### Statistical analyses

The ‘survival’ R package (2.41.3) was used for Kaplan-Meier curve plotting and statistical analysis of overall survival (OS) based on mRNA expression of SeCEP genes among TCGA BRCA samples, and distant relapse free survival (DRFS) for the GSE25066 samples.). [32] The survival functions surv, survfit, and survdiff were used for Kaplan-Meier estimator and log-rank tests. The survival function coxph was used for Cox proportional hazard survival analysis. The wilcox.test function in R was to perform Mann-Whitney-Wilcoxon Test. All genes in the TCGA (*n* = 18,097) or METABRIC (*n* = 16,555) data sets were included in order to obtain an adjusted *p*-value for multiple comparisons.. The adjusted *p*-values were calculated by submitting all the p-values from the individual Mann-Whitney-Wilcoxon Tests determined for each gene to the p.adjust function in R using the ‘fdr’ method.

## Results

### The secreto-transcriptomic workflow for discovering candidate biomarkers for non-invasive, individualized prognosis

**Fig. 1:**
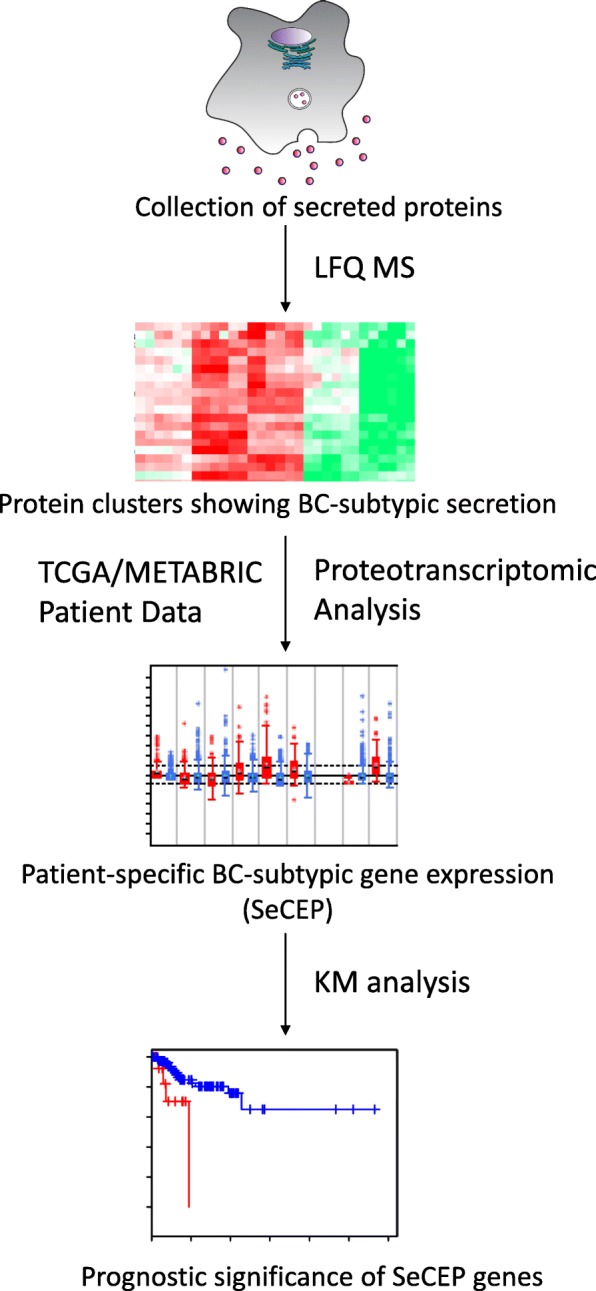
Schematic of secreto-transcriptomic approach for identifying putative liquid biopsy prognostic markers. Label-free quantitative (LFQ) proteomics is used to identify differentially secreted proteins from different BC subtypes. Proteotranscriptomic analysis of TCGA/METABRIC patient data identifies luminal or basal genes showing Secretion-Correlated mRNA Expression Pattern (SeCEP). Kaplan-Meyer (KM) analysis then identifies unique combinations of SeCEP genes showing mRNA co-overexpression patterns of prognostic significance

As shown in Fig. 1, LFQ proteomics was first used to determine the compositional differences in the secretomes isolated from different BC subtypes versus non-malignant cells. The proteins showing BC or PAM50 subtype-specific or subtypic secretion were identified by LFQ-based Perseus analysis. [33] Taking advantage of the databases of two large patient cohorts, TCGA [28] and METABRIC (Molecular Taxonomy of BC International Consortium) [29], which contain clinic-pathologically correlated gene-expression or transcriptomic data, we retrospectively established the proteo-transcriptomic links between BC subtypically secreted proteins and the patient-specific mRNA-expression alterations of the genes that encode these proteins. As a result, this proteo-transcriptomic approach identified the secretome-encoding genes that showed a secretion-correlated mRNA expression pattern (SeCEP), wherein the patient-specific mRNA expression of these genes was positively correlated with increased secretion of the proteins encoded by these genes in similar BC subtypes. This expression-to-secretion correlation not only indicated those genes that are fully translated into extracellularly functional, oncogenically active proteins, but also identified new phenotypic markers that describe the similar PAM50-classified BC subtype. Further, Kaplan-Meier (KM) survival analyses were used to distinguish*,* from *>* 18,000 genes, patient-specific mRNA expression patterns of select SeCEP genes of prognostic value, indirectly identifying those proteins showing BC subtypic secretion as candidate markers for non-invasive prognostic prediction.

### LFQ secretome screening identified particular protein clusters showing BC-subtypic secretion

We used a similar LFQ proteomic approach [34] to comparatively profile the extracellular proteins secreted from different cell lines, respectively representing the BLBC/TNBC subtypes (MDA-231 and HCC1806), luminal subtypes (MCF-7 and T47D), and non-malignant mammary control (MCF10A). (Additional file 1: Figure S1a) A total of 2345 proteins were identified in these five cell lines, and the numbers of the secreted proteins identified in each cell line were given in Additional file 9: Table S1. Using existing databases of secreted proteins we then examined the purity of our secretome isolation. The analysis of Gene Ontology Cellular Component (GOCC) indicated that 685 or 29% of the total identified proteins were previously known for their locations in the extracellular space or plasma membrane. Also, 503 proteins were previously known as secreted or highly likely secreted proteins in the MetazsecKB database that is generated by multiple bioinformatics tools including SignalP4, TMHMM, and TargetP. [35] By comparing our identifications to a number of experimentally identified secretomes 832 proteins were found in common in the secretome from the LPS-stimulated macrophages [34] and 1042 proteins were also identified in a breast cancer secretome. [36] These results in combination validated the high quality of our secretome preparation and analysis.

To identify the proteins showing BC-subtypic secretion we used the Profile Plot function of the Perseus software platform [33] to determine the relative abundances of individual secreted proteins across different cell lines, which correlate with the LFQ ratios of identified proteins. Profile Plot performs pattern matching but does not perform statistical testing on the identified proteins, therefore the statistical significance of protein abundance changes between BC subtypes was validated by one-way ANOVA. As a result we identified clusters that contain the proteins showing increased or decreased secretion only in either BLBC- or luminal-subtypic cell lines, respectively. (Additional file 1: Figure S1 b-e).

**Fig. 2:**
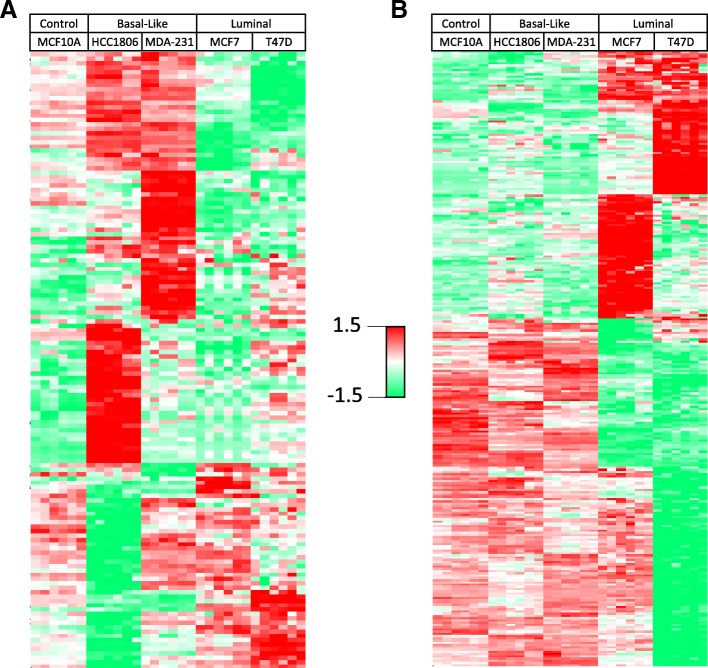
A heatmap of unsupervised hierarchical clustering analysis of the z-scored basal-specific (**a**) and luminal-specific (**b**) proteins (rows) secreted by five cell lines (columns). Each cell line is represented by 3 biological replicates and 2 technical replicates. Red indicates higher secretion, green indicates lower secretion, and white indicates mean secretion

For example, we identified 55 proteins as having BLBC-specific secretion in both BLBC cell lines, including 35 proteins with increased secretion and 20 proteins with decreased secretion compared to the luminal and non-malignant control cell lines. Meanwhile, there were 86 additional proteins showing either increased or decreased secretion in one of the BLBC cell lines. (Fig. 2a; Additional file 10: Table S2) In view of BC-related function of these BLBC-specifically secreted proteins, several factors involved in tumor progression and metastasis showed increased secretion, including CD44, [37] heat shock protein family A member 5 (HSPA5), [38] and heat shock protein 90 beta family member 1 (HSP90B1). [39] Meanwhile, some proteins such as E-cadherin (CDH1) and damage-specific DNA binding protein 1 (DDB1) that were known to be down-regulated in BLBC [40, 41] showed BLBC-specific reduction in secretion.

Similarly, we identified a total of 274 proteins that showed luminal-specific secretion changes in one or both of the luminal cell lines (Fig. 2b; Additional file 11: Table S3), including decreased secretion of several members of the cathepsin family of globular proteases such as CTSB, CTSL, and CTSZ that were known to promote breast cancer progression and metastasis, [42] and the increased secretion of metastasis suppressor PEBP1 (a.k.a. RKIP) that showed luminal-specific intracellular expression. [43]

Immunoblotting of some of BC-subtypic secreted proteins showed consistent results with LFQ secretome screening (Additional file 2: Figure S2).

Using bioinformatics tools including Ingenuity Pathway Analysis (IPA) [22] and the Search Tool for the Retrieval of Interacting Genes/Proteins (STRING) database [23] we then studied the biological processes and pathways in which these BC-subtypic secreted proteins are involved. In the IPA annotation, greater than 95% of all identified BC-subtypic secreted proteins, i.e.*,* 136 of 141 BLBC-specific and 269 of 274 luminal-specific secreted proteins were respectively cancer-related.

Additional file 3 Figure S3a shows the biological processes that are over-presented by the BLBC- and luminal-specific proteins. Although major biological processes are comparable between subtypes, more detailed analysis of these broad categories highlighted the differences between subtype characteristics. BLBC-specific proteins were involved in increased cell movement or migration, invasiveness of breast cancer cells, and cell survival, while luminal-specific proteins were associated with decreases in cell movement and vascularization, indicating the aggressiveness differences between these two BC subtypes (Additional file 3: Figure S3b).

IPA analysis of BLBC-specific secreted proteins (Additional file 3: Figure S3c) indicated the activation of a few known BC-driving signaling pathways, including PI3K-Akt signaling, [44] protein kinase A signaling, [45] signaling by Rho family GTPases, [46] the 14–3-3-mediated signaling associated with BC oncogenesis, [47] and the actin cytoskeleton signaling involved in the epithelial-mesenchymal transition (EMT). [48] Meanwhile, the altered secretion of other BLBC-specific proteins indicated that the activity of the HIPPO signaling was suppressed in BLBC cells, which could lead to a more invasive tumor phenotype. [49] On the other hand, the luminal-specific secreted proteins revealed activation of HIPPO and mTOR signaling along with the suppression of eIF2 signaling, G2/M DNA damage checkpoint regulation, and ILK signaling (Additional file 3: Figure S3c).

To further determine the functional networks involving BC subtypic secreted proteins we performed protein-protein interaction (PPI) analysis using STRING, which revealed statistically significant enrichment of PPIs among the proteins secreted in both BLBC-specific (*p* < 1e-16) and luminal-specific (*p* < 1e-16) manners (Additional file 4: Figure S4). The Gene Ontology Biological Process enrichment of the proteins with BLBC-specific increased secretion identified multiple subnetworks associated with protein folding, regulation of cell communication, regulation of apoptosis, cell development, regulation of cell motility, blood coagulation, and proteolysis. Analysis of the proteins with decreased secretion in BLBC cells also revealed particular subnetworks/pathways with suppressed activities, including DNA damage response, regulation of actin depolymerization, and regulation of cell-cell adhesion. In contrast, the proteins showing luminal-specific increases of secretion over-represented the subnetworks associated with regulation of growth, and cell differentiation while the proteins with decreased secretion in luminal cells were involved in positive regulation of apoptotic process, angiogenesis, extracellular matrix disassembly, and cell motility. These results showed that the proteins secreted or secretomes are characteristic of distinct BC subtypes.

### The genes that encode increasingly secreted proteins showed secretion-correlated mRNA over-expression patterns in BC patients in a PAM50-subtypic manner

To determine the clinicopathological relevance of the proteins showing BC subtypic secretion, in the databases of TCGA [28] and METABRIC [29] we retrospectively examined patient mRNA expression patterns for the genes that encode the proteins showing either BLBC- or luminal-specific secretion. These databases contain large cohorts of > 2600 BC patients that were classified by PAM50 as the BLBC/TNBC, luminal-A and luminal-B, Her2+, and normal-like BC subtypes, and the information about mRNA expression, mutations, copy-number variations, and associated clinical/pathological data (stages/grades and relapse status).

First, to determine the mRNA expression differences between BLBC and luminal A/B TCGA patients we performed a Mann-Whitney-Wilcoxon Test on the z-scored expression values (downloaded from the cBioPortal for Cancer Genomics [25, 26]) of the two PAM50-subtypic populations. This test was performed on all secreted protein-encoding genes in each subtype-specific dataset, and *p*-values were adjusted by the Benjamini Hochberg procedure for multiple testing. Using this multi-testing scheme, a secreted protein-encoding gene was classified as BLBC if the expression level between the two PAM50-subtypic populations was significantly different (adjusted p-value < 0.05) and the median mRNA expression was greater among BLBC patients. Likewise, a gene was classified as luminal if its median mRNA expression level was higher for luminal patients and the gene showed a statistically significant difference between luminal and BLBC patients (adjusted p-value < 0.05).

**Fig. 3:**
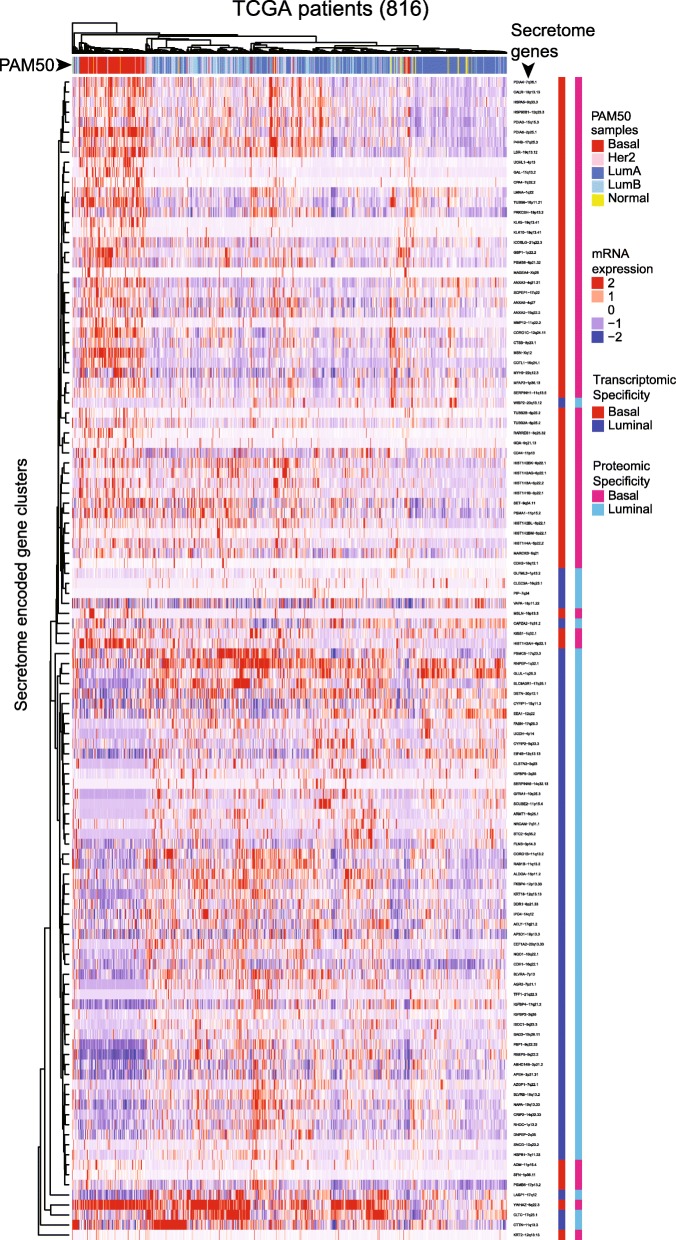
Heatmap of the unsupervised hierarchical clustering of mRNA expression of SeCEP genes (rows) in the TCGA patient cohort (columns). The PAM50 subtype of each patient is indicated by the row above the heatmap. Proteomic (left) and transcriptomic (right) subtype-specificity are indicated by the columns to the right of the heatmap. Within the heatmap red represents higher expression, blue represents lower expression, and white represents mean expression

**Fig. 4:**
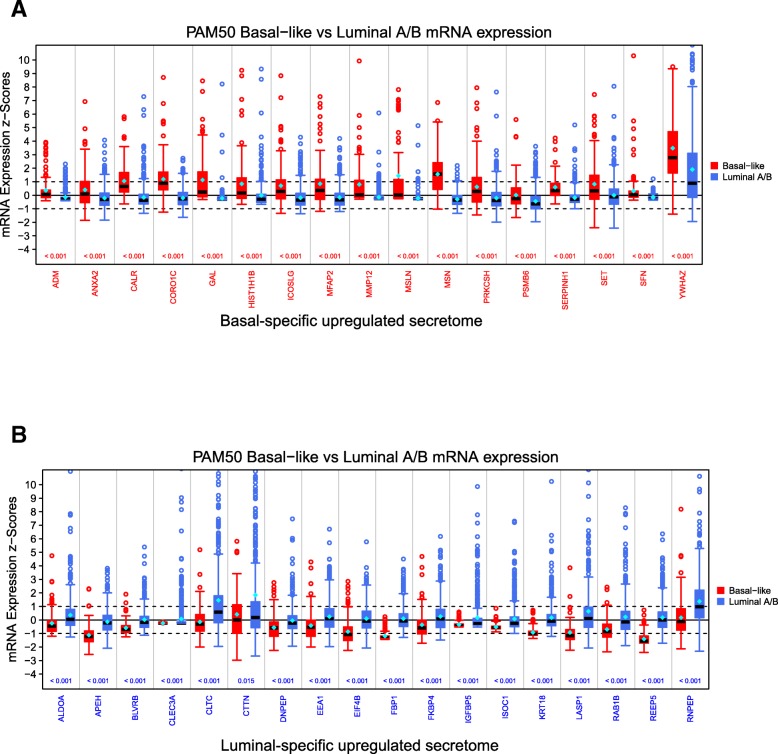
Box plots showing the statistically significant altered mRNA expression (x-axis) for **a** BLBC and **b** luminal secreted proteins among TCGA patients. The distribution of mRNA expression for BLBC patients is shown in red on the left, and for luminal A/B patients in blue on the right. The z-scored mRNA expression is displayed on the y-axis. Values > 1 are considered to be significantly up-regulated, values < − 1 to be significantly downregulated, and values between − 1 and 1 are considered “not altered”. The median value is displayed as a black bar inside the box. A Mann-Whitney-Wilcoxon Test to ascertain the expression differences between the two PAM50-subtypic populations was performed. The *p*-value is displayed above the x-axis with *p*-values < 0.05 colored red if expression is higher for BLBC and blue if expression is higher for luminal samples

On a systems view, a heat map of unsupervised hierarchical clustering showed patient mRNA expression patterns for those genes that encode the proteins demonstrating PAM50-subtypic secretion in TCGA patients (Fig. 3). In a statistically significant manner, we identified a secretome-to-patient transcriptome or secreto-transcriptomic link for some genes that encode PAM50-subtypic secreted proteins, i.e., we found a secretion-correlated mRNA overexpression pattern or SeCEP wherein the PAM50-subtypic secretion of some proteins showed *cis*-mRNA expression of their encoding genes in patients with the corresponding PAM50-subtypes (Fig. 4). For example, mRNA overexpression of 57 genes that encode BLBC-specific secreted proteins clustered BLBC patients while 60 genes that encode luminal-specific proteins with increased secretion showed mRNA overexpression in luminal patients. These results indicated that these secretome genes are fully translated into the oncogenically active secretome in a BC-subtypic manner.

Further, we observed interpatient heterogeneity in the mRNA expression pattern of secretome genes within each PAM50 subtype, i.e., not all SeCEP genes were simultaneously overexpressed at the mRNA level in each individual BLBC or luminal patient. Bearing in mind that mRNA expression patterns of PAM50 genes are insufficient to stratify the patient subsets with different clinical outcomes or prognoses, we reasoned that, within a single PAM50-classified subtype, these patient mRNA expression variations of select SeCEP genes can mark the patient subpopulations with distinct prognoses.

### Patient-specific mRNA co-overexpression patterns of select secretome-encoding genes mark the high-risk subpopulations of PAM50-subtypic patients with poor prognosis

To identify BC-subtypic secreted proteins of prognostic significance, we performed Kaplan-Meier (KM) analysis on PAM50-subtypic patients in the two independent datasets TCGA and METABRIC [29, 30] for any combination of up to five SeCEP genes having mRNA overexpression (z-score > median z-score) for all genes in the combination. The statistical significance of each gene combination was determined by a multi-parameter threshold including log-rank *p* value < 0.05 and lower 95 confidence interval for the hazard ratio > 1 in both the TCGA and METABRIC datasets.

**Fig. 5:**
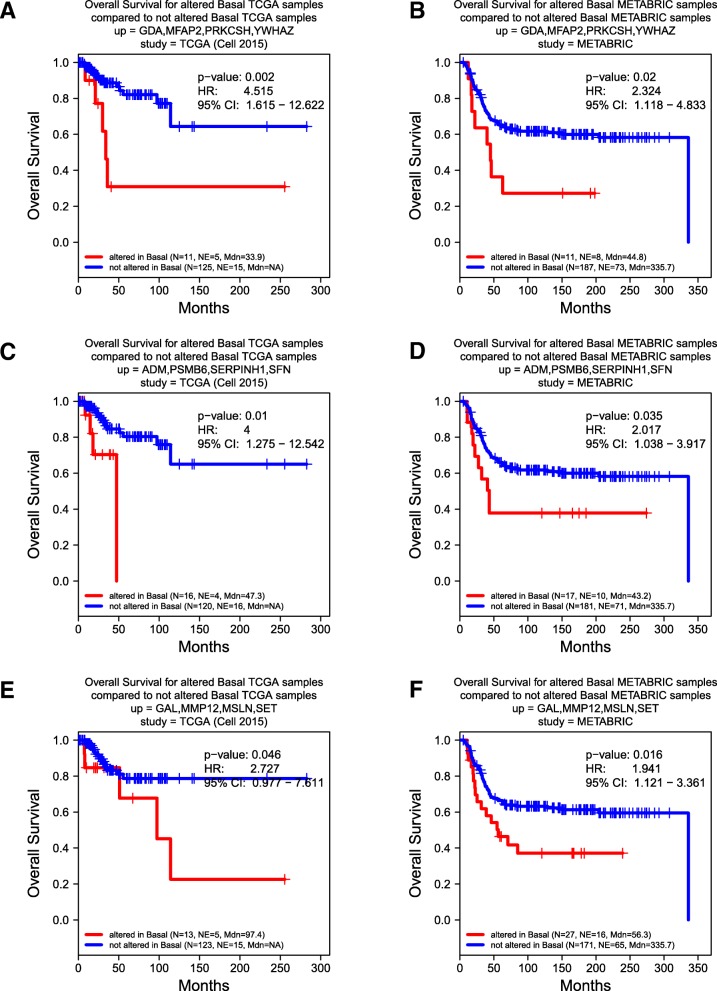
Correlation between Kaplan-Meier survival plots of the clinical outcomes and mRNA co-overexpression of indicated basal SeCEP genes based on TCGA (**a**, **c**, **e**) and METABRIC (**b**, **d**, **f**) patient data. “N” refers to “Number of patients,” and “NE” refers to “Number of Events (Overall Survival status = DECEASED)”. Each plot shows the log-rank p-value and Hazard Ratio (HR) with 95% Confidence Interval (CI) between the two groups. The red line designates the patient subpopulation showing statistically significant overexpression of the indicated basal-specific genes (“altered”). The blue line designates the group of patients not showing statistically significant overexpression of the indicated basal-specific genes (“not altered”)

For example, we identified subpopulations of approximately 8% or more BLBC patients who showed mRNA co-overexpression of four BLBC-specific SeCEP genes, YWHAZ, GDA, MFAP2, and PRKCSH in correlation with poor survival (Fig. 5a,b). YWHAZ, which encodes the 14–3-3ζ protein, was characterized as a promoter of cell survival which, when overexpressed, is associated with poor prognosis and disease-free survival. [50, 51] Another SeCEP gene combination indicating the co-overexpression-correlated poor prognosis was ADM, PSMB6, SERPINH1, and SFN (Fig. 5c,d). ADM was known to promote angiogenesis, cell survival, and metastasis, [52, 53] and was associated with poor prognosis in ovarian cancer patients. [54] Interestingly, although SFN (14–3-3σ or stratifin) was considered as a tumor suppressor, overexpression in BLBC was reported. [55] Recently, overexpression of SFN was found to be associated with tumor invasion and migration. [56] Another BLBC subpopulation showed co-overexpression of GAL, MMP12, MSLN, and a multifunctional oncoprotein SET [57] (Fig. 5e,f).

**Fig. 6:**
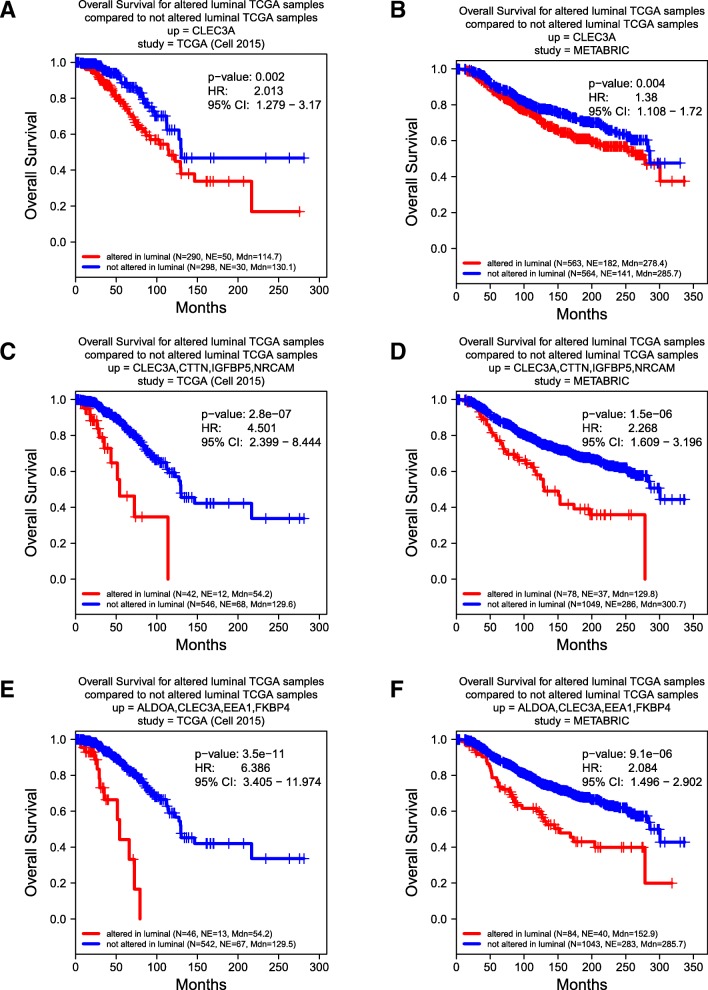
Correlation between Kaplan-Meier survival plots of the clinical outcomes and mRNA co-overexpression of indicated luminal SeCEP genes based on TCGA (**a**, **c**, **e**) and METABRIC (**b**, **d**, **f**) patient data. “N” refers to “Number of patients,” and “NE” refers to “Number of Events (Overall Survival status = DECEASED)”. Each plot shows the log-rank p-value and Hazard Ratio (HR) with 95% Confidence Interval (CI) between the two groups. The red line designates the patient subpopulation showing statistically significant overexpression of the indicated luminal-specific genes (“altered”). The blue line designates the group of patients not showing statistically significant overexpression of the indicated luminal-specific genes (“not altered”). Overexpression of CLEC3A provides less prognostic value than overexpression of CLEC3A in combination with other secreted factors

Similarly, this secreto-transcriptomic approach enabled identifications of the distinct subpopulations of luminal patients with poor prognosis. Further, as an example of how the co-overexpression of multiple SeCEP genes improves the specificity and sensitivity in predicting personalized prognosis, as shown in Fig. 6a,b, overexpression of CLEC3A alone indicated modest differences in the overall survival rate of two major luminal patient subpopulations. However, the luminal patient subsets showing co-overexpression of CLEC3A with CTTN, IGFBP5, NRCAM were statistic-significantly correlated with worse prognosis and can be readily discriminated from other luminal patients (Fig. 6c,d). Among these SeCEP genes, CLEC3A is a C-type lectin that promotes tumor adhesion in breast cancer [58] and was recently found to enhance plasminogen activation by tissue-type plasminogen activator. [59] CTTN encodes cortactin, an actin cytoskeleton regulator that promotes metastasis in breast cancer. [60] Meanwhile, co-overexpression of CLEC3A with ALDOA, EEA1, and FKBP4 was also associated with substantially worse prognosis than CLEC3A alone (Fig. 6e,f).

Importantly, the use of co-overexpressed SeCEP genes can further resolve individual luminal subtypes among luminal patients to identify the high-risk subpopulations of luminal-A or luminal-B patients. For example, the luminal-A subpopulations overexpressing CAPZA2, FKBP4, KRT18, and OLFML3 exhibited poor prognosis with decreased overall survival, but this combination did not distinguish any subpopulation of luminal-B patients or combined luminal A/B groups (Additional file 5: Figure S5 a,b). Also, co-overexpression of FASN, IGFBP5, ISOC1, and PIP was likewise specific to the luminal-A group (Additional file 5: Figure S5 c,d). Interestingly, although each of these genes has been reported to play a role in breast cancer development, progression, or metastasis, [61–64] over-expression of these genes individually did not provide subtype-specific prognostic value. Overall, we found 52 gene combinations with co-overexpression that showed poor survival among luminal-A patients but not in other BC subtypes.

Several gene co-overexpression patterns specifically correlated with luminal-B patient prognosis were also identified. In the co-overexpression pattern involving HSP90B1, EEF1A2, EIF4B, and KRT18, (Additional file 6: Figure S6 a,b) HSPB1 was known to play a role in epithelial-mesenchymal transition and tumors overexpressing HSPB1 demonstrated enhanced drug resistance. [65] Similarly, luminal-B patients overexpressing a combination of AGR2, CYFIP2, KRT18, and RAB1B exhibited worst overall survival while luminal-A patients and the combined luminal A/B group showed no significant differences in survival (Additional file 6: Figure S6 c,d). In total we identified 39 gene combinations that, when overexpressed, indicated poor overall survival specifically among luminal-B patients. Our combined results demonstrated that patient-specific co-overexpression of SeCEP genes can resolve the interpatient heterogeneity within different PAM50-subtypes, confirming that these gene expression alteration patterns are prognostically meaningful in distinguishing the subsets of BLBC or luminal patients with distinct clinical outcomes with multi-testing of large patient cohorts.

Notably, unsupervised hierarchical clustering mRNA expression of luminal and basal SeCEP genes using the TCGA patient cohort revealed that HER2-overexpressing or -enriched patients did not cluster together but were interspersed among primarily luminal A/B patients. (Fig. 3) This result implied that various clinical outcomes of HER2-enriched patients could be represented by select luminal SeCEP genes. We therefore searched for altered mRNA expression patterns of luminal SeCEP genes in correlation with destinct clinical outcomes of HER2+ patients. Generally, patients with the HER2-enriched subtype show overall poor survival similar to BLBC patients. [66] In KM analysis, we identified unique gene combinations associated with poor survival among HER2-enriched patient subpopulations which were not prognostically significant among luminal patients. For example, HER2-enriched patients showing co-overexpression of CAPZA2, CBX1, G6PD, and NQO1 had worse survival (Additional file 7: Figure S7 a,b). NQO1 was highly expressed in BC patients with high HER2 expression and was linked to increased metastasis. [67] High expression of CYFIP1, DDR1 and GYG1 was also associated with worse survival (Additional file 7: Figure S7 c,d), and DDR1 was linked to BC invasion and drug resistance. [68, 69] Another HER2-enriched subpopulation with poor survival showed co-overexpressed G6PD, CYFIP1, PSMC2, and KYNU (Additional file 7: Figure S7 e,f), the latter of which has been implicated in increased metastasis and tumor aggressiveness. [70] In sum, these results indicate that altered mRNA expression patterns of select luminal SeCEP genes can be used to distinguish the distinct subpopulations of HER2-enriched patients with poor prognosis.

**Fig. 7:**
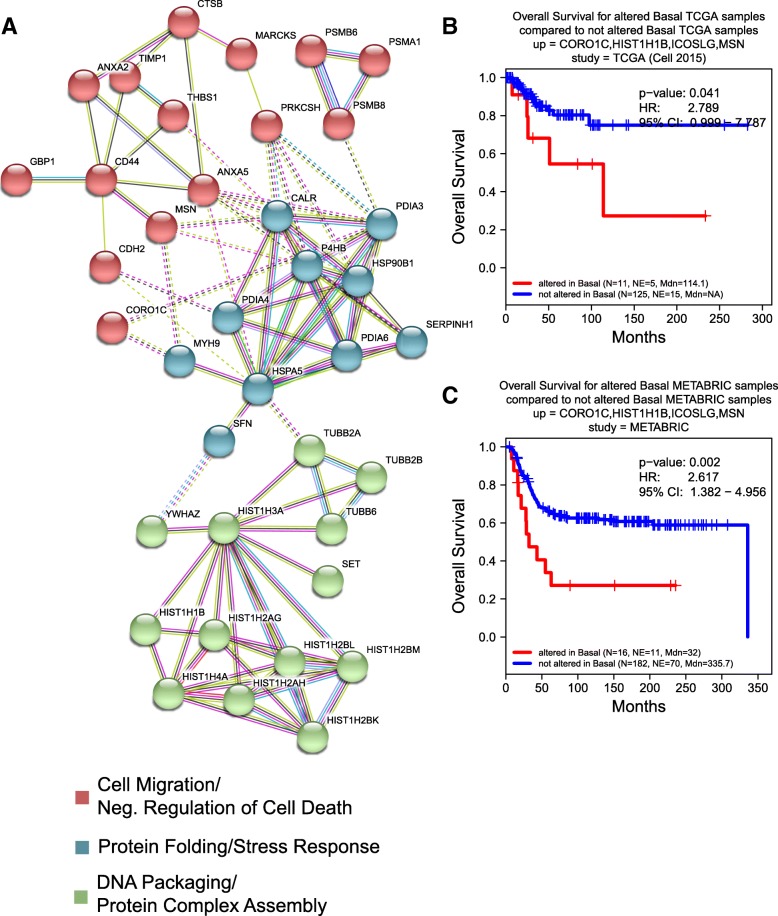
**a** The interactive subnetworks of basal SeCEP genes. B,C) Correlation between Kaplan-Meier survival plots of the clinical outcomes and mRNA co-overexpression of indicated luminal SeCEP genes based on TCGA (**b**) and METABRIC (**c**) patient data. “N” refers to “Number of patients,” and “NE” refers to “Number of Events (Overall Survival status = DECEASED)”. Each plot shows the log-rank p-value and Hazard Ratio (HR) with 95% Confidence Interval (CI) between the two groups. The red line designates the patient subpopulation showing statistically significant overexpression of the indicated basal-specific genes (“altered”). The blue line designates the group of patients not showing statistically significant overexpression of the indicated basal-specific genes (“not altered”). Three of the four overexpressed genes are members of basal-specific interactive subnetworks

More importantly, the majority of the genes encoding BLBC- or luminal-specific secretome in networks showed statistically significant, secretion-correlated cis-mRNA expression in some BC patients. Further, by identifying their co-overexpressed patterns in BC-subtypic patients, we revealed the pathological or prognostic significance of these secreted proteins in multiple interactive sub-networks (Fig. 7a). Strikingly, the majority of the BLBC-specific proteins involved in the interactive subnetworks were associated with unfolded protein response, cell migration, and negative regulation of cell death. Specifically, the glycoprotein THBS1 promoted BC invasion and metastasis and was associated with disease recurrence in BC patients. [71, 72] Similarly, higher serum levels of metallopeptidase inhibitor TIMP-1 were associated with increased likelihood of BC metastasis. [73] Further, the disulfide isomerase PDIA6 promoted tumor immune evasion [74] and enhanced cell proliferation by activating Wnt/β-catenin signaling. [75] Three of four genes in the combination of CORO1C, MSN, ICOSLG, and HIST1H1B are in this subnetwork, and KM analysis reveals a significant decrease in the overall survival rate of BLBC patients overexpressing these genes (Fig. 7b,c).

**Fig. 8:**
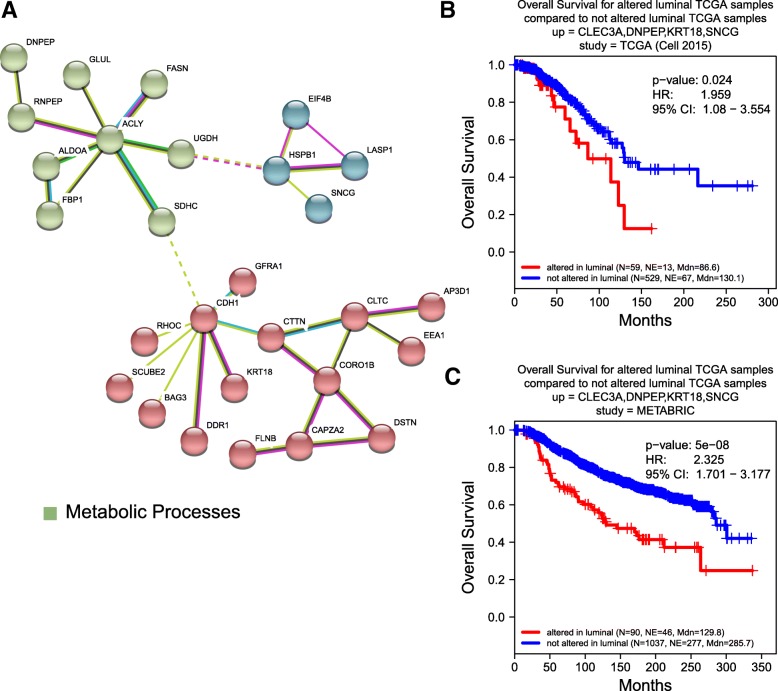
**a** The interactive subnetworks of luminal SeCEP genes. B,C) Correlation between Kaplan-Meier survival plots of the clinical outcomes and mRNA co-overexpression of indicated luminal SeCEP genes based on TCGA (**b**) and METABRIC (**c**) patient data. “N” refers to “Number of patients,” and “NE” refers to “Number of Events (Overall Survival status = DECEASED)”. Each plot shows the log-rank p-value and Hazard Ratio (HR) with 95% Confidence Interval (CI) between the two groups. The red line designates the patient subpopulation showing statistically significant overexpression of the indicated luminal-specific genes (“altered”). The blue line designates the group of patients not showing statistically significant overexpression of the indicated luminal-specific genes (“not altered”). Three of the four overexpressed genes are members of luminal-specific interactive subnetworks

Luminal-specific subnetworks were also identified, (Fig. 8a) however there was no biological process enrichment observed. These oncogenically active interacting proteins included BAG3 which reduced BC cell adhesion and increased motility. [76] This network also included RHOC, a small GTPase that regulates cytoskeletal architecture [77] and is associated with increased rates of metastasis. [78] SNCG, a neuronal protein overexpressed in BC was also associated with higher likelihood of metastasis. [79] TCGA and METABRIC patients exhibiting a four gene co-overexpression pattern involving SNCG, CLEC3A, DNPEP, and KRT18, three of which are members of the interacting subnetworks, had lower overall survival rates (Fig. 8b,c). Together, these results indicate the coordinated, extracellular oncogenic activity of the networked proteins.

### Secreto-transcriptomic analysis identified patient-specific co-overexpression patterns of select secreted proteins as prognostic markers to predict personalized response to therapy

Nether the TCGA nor the METABRIC study was designed to answer specific clinical questions. To assess the clinical significance of altered mRNA expression of multiple SeCEP genes in predicting the response to specific therapeutic interventions, we next looked for distinct combinations of SeCEP genes showing statistically significant changes in distant relapse free survival (DRFS) among patients receiving neoadjuvant taxane-anthracycline therapy in the clinical trial GSE25066. [80] Following similar procedures to those described above for TCGA and METABRIC, we performed KM analysis on the BLBC-SeCEP genes in combinations of up to five genes having mRNA overexpression (z-score > median z-score) for all genes in the combination.

**Fig. 9:**
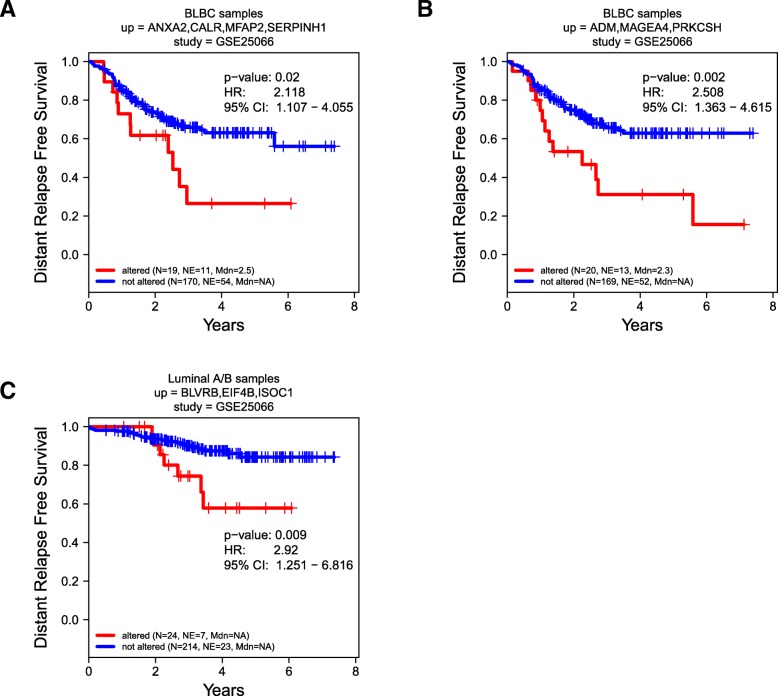
Correlation between Kaplan-Meier survival plots of the clinical outcomes and mRNA co-overexpression of indicated basal (**a**, **b**) or luminal (**c**) SeCEP genes based on GSE25066 patient data. “N” refers to “Number of patients,” and “NE” refers to “Number of Events (Distant Relapse Free Survival status = 1)”. Each plot shows the log-rank p-value and Hazard Ratio (HR) with 95% Confidence Interval (CI) between the two groups. The red line designates the patient subpopulation showing statistically significant overexpression of the indicated subtype-specific genes (“altered”). The blue line designates the group of patients not showing statistically significant overexpression of the indicated subtype-specific genes (“not altered”). See also Additional file 8: Figure S8

Among BLBC patients, we found 12 combinations with > 10% of both GSE25066 and TCGA patients overexpressing each gene in the combination and having a significant difference in DRFS. (Additional file 12: Table S4). Examples are shown in Fig. 9a,b. One such combination was ANXA2, CALR, MFAP2, and SERPINH1. ANXA2 has been reported as an independent predictor of poor prognosis in breast cancer patients receiving neoadjuvant therapy, [81] however overexpression of ANXA2 alone did not have a statistically significant impact on DRFS among GSE25066 patients. Likewise, co-overexpression of ADM, MAGEA4, and PRKCSH was also associated with a statistically significant change in DRFS. Similar analysis of luminal-SeCEP gene combinations yielded five combinations with at least 10% of patients in both the GSE25066 and TCGA datasets overexpressing all genes in the combination and *p* value < 0.05. (Additional file 13: Table S5) One combination was BLVRB, EIF4B, and ISOC1 (Fig. 9c). Importantly, BLVRB is associated with the development of chemotherapeutic resistance, though overexpression of BLVRB alone did not predict worse patient outcomes. [82]

Overall, these analyses identified the subpopulations within each PAM50 subtype with resistance to neoadjuvant anthracycline-taxane therapy along with the correlation to their poorer overall survival. Thus, we demonstrate the potential clinical uses of the analysis to aid the clinician in determining the appropriate therapeutic intervention to be employed.

## Discussion

The development of a novel secreto-transcriptomic approach underlies our innovation in the identification of liquid biopsy biomarkers capable of discriminating between patient subpopulations having variable outcomes. Recognizing that single-omics approaches are insufficient for making these distinctions, due to the negligible data on the oncogenic phenotype provided by genomics/transcriptomics and the inadequate phenotypic coverage of patient-specific alterations proteomics offers, our secreto-transcriptomic workflow is a multi-omic integrated method which offers a robust and efficient scheme to distinguish patient subpopulations within each BC subtype. First, by using a label-free quantitation (LFQ)-based nanoLC-MS/MS approach for secretome profiling, [34] we comparatively analyzed the compositional differences in the extracellular proteins secreted from a series of BC cell lines representing various BC- or PAM50-subtypes. We then determined the clinicopathological relevance of the proteins showing subtype-specific or subtypic secretion by a retrospective proteo-transcriptomic analysis [20] of the BC patient datasets (> 2600 patients) from The Cancer Genome Atlas (TCGA) [28] and METABRIC (Molecular Taxonomy of BC International Consortium). [29] We found a PAM50-subtypic Secretion-Correlated mRNA Expression Pattern (SeCEP) wherein the PAM50-subtypic secretion of some proteins showed statistically significant *cis*-mRNA expression of the genes that encode them in patients with the corresponding PAM50-subtypes. This expression-to-secretion correlation highlighted those genes that are fully translated into the oncogenically active secretome in a PAM50-subtypic manner. Further, we observed that patient-to-patient mRNA expression variations of individual secretome genes describe the interpatient heterogeneity within each single PAM50 subtype. In this regard, patient-specific co-overexpression of distinct SeCEP genes were found in correlation with specific prognoses within distinct subsets of BLBC or luminal-A and luminal-B patients. Currently, available blood-based tests for cancer prognosis or diagnosis are often based on a single gene or protein marker, therefore lacking the specificity and sensitivity in determining individualized clinical outcomes. [83] Our identification of multi-gene or multi-protein panels as systems signatures can precisely describe the predominant tumor phenotype with significantly improved phenotype accuracy. Because our workflow starts with the identification of tumor-phenotypic alterations and work back to the genotypic data with the coverage of patient-specific alterations, we are able to bypass the need for extensive modeling [84] or analysis of a large number of patients [85] by pinpointing a few prognostically significant marker genes.

Our dissection of the BC-subtypic secretomes highlights the differences between subtype-characteristic extracellular functions reflecting the divergent underlying pathologies of each subtype. We found that although both luminal and basal secreted proteins fall into the same broad categories (e.g. cell motility), the functional roles of these proteins are significantly different between subtypes. For example, cellular movement was a highly overrepresented category in the secretomes of both subtypes, but the basal-specific proteins were promotive of cell motility while the luminal-specific proteins were inhibitive, which is in line with the more aggressive nature of BLBC subtype in general. Similarly, pathway activation analysis found the same pathways enriched in both subtypes, but with opposite activation states.

We identified multiple over-secreted proteins exhibiting a BC-subtypic SeCEP consistently in both TCGA and METABRIC databases with > 2600 BC patients which constitute the subtype-specific fully translated oncogenic-active secretome. Additionally, the co-overexpression of multiple SeCEP genes in unique combinations was prognostic of differential survival rates of subpopulations within each PAM50-subtype. Further, these co-overexpressed gene combinations were distinct for each subtype, i.e. combinations showing decreased overall survival in one subtype did not exhibit altered survival rates in other subtypes.

There are some important considerations to note in this study. First, due to the significant breast tumor heterogeneity our work cannot, and is not intended to, identify all of the secreted proteins relevant to the characteristics of a given tumor. We used breast cell lines as a model system to identify potential markers and reinforce these identifications with a broad set of patient data. In order to efficiently connect patient outcomes to potential markers, we must select practical criteria. The high expression/high secretion correlation provides a reasonable and straightforward link between the secretome and the transcriptome. Alternate expression/secretion patterns are observed, however these are harder to quantify and correlate. Importantly, it is not necessary to identify every gene combination of relevance in order to identify specific patient subpopulations with poorer outcomes. The present study also has some limitations which preclude the ability to identify all such subgroups, including the number of basal and luminal cell lines examined and the exclusion of the HER2-enriched subtype from the secretome analysis. Despite these limitations our work names several noteworthy gene combinations which define specific patient subpopulations, but more importantly provides a template for the further identification of combinations defining other subgroups.

Because multiple SeCEP genes showing prognostically-significant mRNA co-overexpression in a marker panel were identified as over-secreted proteins in a single BC subtype, these gene-coded proteins are putative liquid biopsy markers to distinguish high-risk populations within PAM50-subtypic classification. Importantly, we also demonstrated the clinical utility of this method in identifying patient subpopulations with the worst outcomes in response to specific therapeutic interventions.

## Conclusions

In summary, our novel secreto-transcriptomic method efficiently and precisely delineated high-risk subpopulations within each PAM50-subtype by linking oncogenically secreted proteins to patient-specific transcriptomic alterations that correlate with distinct clinical outcomes. This multi-omics approach leverages the discrimination of a few tumorigenic/oncogenic alterations in broad transcriptomic profiles of > 18,000 genes, which provide an advantage over any single omics approaches. These multi-gene prognostic markers offer individualized specificity and sensitivity which may guide the clinician to optimize the treatment plan for distinct patient subsets in blood test.

## Additional files

Additional file 1: **Figure S1.** A) A heatmap of unsupervised hierarchical clustering analysis of z-score normalized protein secretion by one non-malignant control (MCF10A), two basal breast cancer (MDA-231 and HCC1806), and two luminal breast cancer (MCF7 and T47D) cell lines highlights intra-subtype heterogeneity. Each cell line is represented by 3 biological replicates and 2 technical replicates. Red indicates higher secretion, green indicates lower secretion, and white indicates mean secretion. Profile plots of 4 representative clusters are shown which demonstrate proteins B) higher in BLBC, C) lower in luminal BC, D) lower in BLBC, and E) higher in luminal BC. Each line represents one protein and the color indicates the density of proteins with similar expression levels. (PPTX 170 kb)

Additional file 2: **Figure S2.** Western blot validation of LFQ data. (PPTX 1693 kb)

Additional file 3: **Figure S3.** A) Over-represented biological processes in the basal-like and luminal BC subtypes. Bars representing the negative log *p*-values of BLBC process enrichment are displayed in orange and luminal values are in blue. B) Biological functions activated (positive z-score) or suppressed (negative z-score) in the BLBC (orange) and luminal (blue) PAM50 subtypes. C) Pathway activation analysis of PAM50 subtypes. Orange bars represent BLBC values and blue bars represent luminal values. Positive z-scores indicate pathway activation; negative z-scores indicate pathway suppression. (PPTX 53 kb)

Additional file 4: **Figure S4.** Protein-protein interaction analysis of A) basal-specific and B) luminal-specific secreted proteins. (PDF 6934 kb)

Additional file 5: **Figure S5.** Correlation between Kaplan-Meier survival plots of the clinical outcomes and mRNA co-overexpression of indicated luminal SeCEP genes based on TCGA (left column) and METABRIC (right column) patient data. “N” refers to “Number of patients,” and “NE” refers to “Number of Events (Overall Survival status = DECEASED)”. Each plot shows the log-rank *p*-value and Hazard Ratio (HR) with 95% Confidence Interval (CI) between the two groups. The red line designates the patient subpopulation showing statistically significant overexpression of the indicated luminal-specific genes (“altered”). The blue line designates the group of patients not showing statistically significant overexpression of the indicated luminal-specific genes (“not altered”). Co-overexpression of distinct sets of genes correlate with statistically significant changes in overall survival in Luminal A patients but not Luminal B or other BC subtypes. (PDF 153 kb)

Additional file 6: **Figure S6.** Correlation between Kaplan-Meier survival plots of the clinical outcomes and mRNA co-overexpression of indicated luminal SeCEP genes based on TCGA (left column) and METABRIC (right column) patient data. “N” refers to “Number of patients,” and “NE” refers to “Number of Events (Overall Survival status = DECEASED)”. Each plot shows the log-rank *p*-value and Hazard Ratio (HR) with 95% Confidence Interval (CI) between the two groups. The red line designates the patient subpopulation showing statistically significant overexpression of the indicated luminal-specific genes (“altered”). The blue line designates the group of patients not showing statistically significant overexpression of the indicated luminal-specific genes (“not altered”). Co-overexpression of distinct sets of genes correlate with statistically significant changes in overall survival in Luminal B patients but not Luminal A or other BC subtypes. (PDF 144 kb)

Additional file 7: **Figure S7.** Correlation between Kaplan-Meier survival plots of the clinical outcomes and mRNA co-overexpression of indicated SeCEP genes based on patient data. “N” refers to “Number of patients,” and “NE” refers to “Number of Events (Overall Survival status = DECEASED)”. Each plot shows the log-rank p-value and Hazard Ratio (HR) with 95% Confidence Interval (CI) between the two groups. The red line designates the patient subpopulation showing statistically significant overexpression of the indicated luminal-specific genes (“altered”). The blue line designates the group of patients not showing statistically significant overexpression of the indicated luminal-specific genes (“not altered”). (PDF 792 kb)

Additional file 8: **Figure S8.** Correlation between Kaplan-Meier survival plots of the clinical outcomes and mRNA co-overexpression of indicated SeCEP genes based on patient data. “N” refers to “Number of patients,” and “NE” refers to “Number of Events (Overall Survival status = DECEASED)”. Each plot shows the log-rank p-value and Hazard Ratio (HR) with 95% Confidence Interval (CI) between the two groups. The red line designates the patient subpopulation showing statistically significant overexpression of the indicated genes (“altered”). The blue line designates the group of patients not showing statistically significant overexpression of the indicated genes (“not altered”). (PDF 121 kb)

Additional file 9: **Table S1.** All proteins LFQR2. (XLSX 644 kb)

Additional file 10: **Table S2.** Basal-Specific Secreted ProteinsR2. (XLSX 52 kb)

Additional file 11: **Table S3.** Luminal-Specific Secreted ProteinsR2. (XLSX 94 kb)

Additional file 12: **Table S4.** Basal GEO + TCGAR2. (XLSX 9 kb)

Additional file 13: **Table S5.** Luminal GEO + TCGAR2. (XLSX 8 kb)

## Abbreviations

BC: Breast cancer
BLBC: Basal-like breast cancer
DRFS: Distant relapse free survival
ER: Estrogen receptor
FDR: False discovery rate
GOCC: Gene ontology cellular component
IPA: Ingenuity Pathway Analysis
KM: Kaplan-Meier
LFQ: Label-free quantitative
METABRIC: Molecular Taxonomy of Breast Cancer International Consortium
MS: Mass spectrometry
nanoLC-MS/MS: Nanoliter liquid chromatography tandem mass spectrometry
OS: Overall survival
PBS: Phosphate buffered saline
PR: Progesterone receptor
SeCEP: Secretion correlated expression pattern
STRING: Search tool for the retrieval of interacting genes/proteins
TCGA: The Cancer Genome Atlas
TNBC: Triple negative breast cancer
