# Supplementary material for: Novel secretome-to-transcriptome integrated or secreto-transcriptomic approach to reveal liquid biopsy biomarkers for predicting individualized prognosis of breast cancer patients
Source: BMC Med Genomics. 2019 May 30;12:78. doi: 10.1186/s12920-019-0530-7 (PMC6543675; doi:10.1186/s12920-019-0530-7)
Supplement: Supplementary file 1 — Figure S1. A) A heatmap of unsupervised hierarchical clustering analysis of z-score normalized protein secretion by one non-malignant control (MCF10A), two basal breast cancer (MDA-231 and HCC1806), and two luminal breast cancer (MCF7 and T47D) cell lines highlights intra-subtype heterogeneity. Each cell line is represented by 3 biological replicates and 2 technical replicates. Red indicates higher secretion, green indicates lower secretion, and white indicates mean secretion. Profile plots of 4 representative clusters are shown which demonstrate proteins B) higher in BLBC, C) lower in luminal BC, D) lower in BLBC, and E) higher in luminal BC. Each line represents one protein and the color indicates the density of proteins with similar expression levels. (PPTX 170 kb) [file 12920_2019_530_MOESM1_ESM.pptx]

## Slide 1
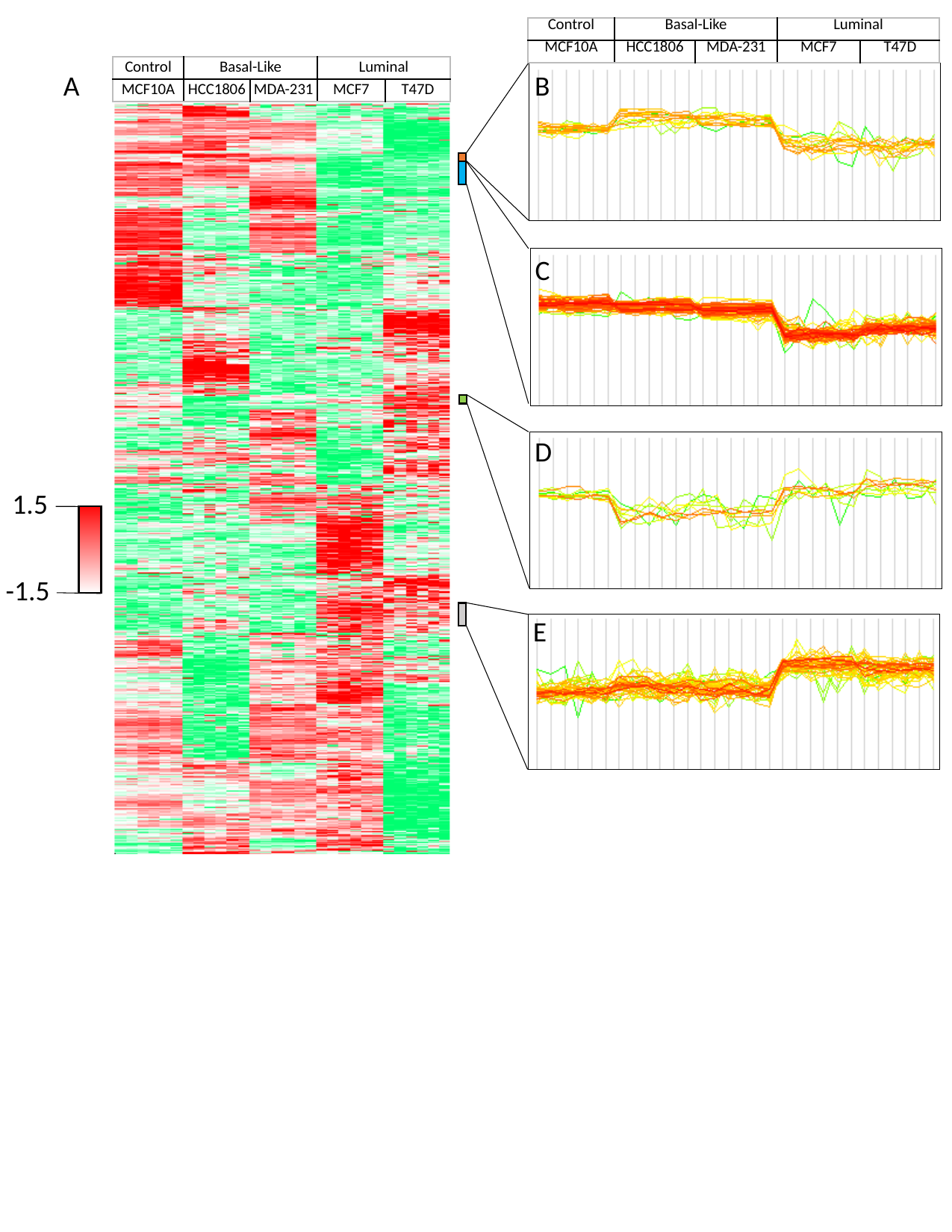

| Control | Basal-Like | | Luminal | |
| --- | --- | --- | --- | --- |
| MCF10A | HCC1806 | MDA-231 | MCF7 | T47D |
| Control | Basal-Like | | Luminal | |
| --- | --- | --- | --- | --- |
| MCF10A | HCC1806 | MDA-231 | MCF7 | T47D |
A
B
C
D
1.5
-1.5
E
