# Supplementary figures and images for: Novel secretome-to-transcriptome integrated or secreto-transcriptomic approach to reveal liquid biopsy biomarkers for predicting individualized prognosis of breast cancer patients
Source: BMC Med Genomics. 2019 May 30;12:78. doi: 10.1186/s12920-019-0530-7 (PMC6543675; doi:10.1186/s12920-019-0530-7)

## Slide 1
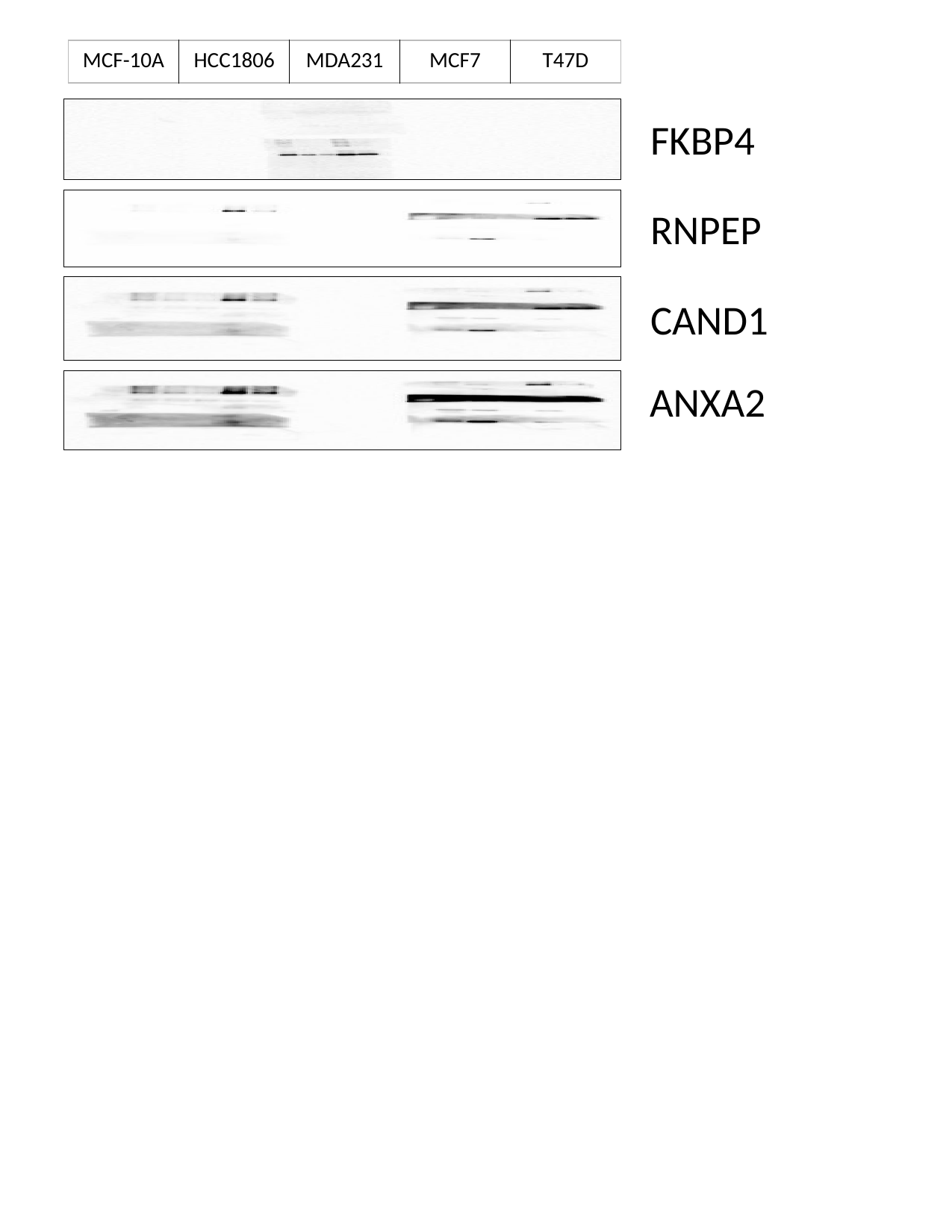

| MCF-10A | HCC1806 | MDA231 | MCF7 | T47D |
| --- | --- | --- | --- | --- |
FKBP4
RNPEP
CAND1
ANXA2

Supplement: Supplementary file 2 — Figure S2. Western blot validation of LFQ data. (PPTX 1693 kb) [file 12920_2019_530_MOESM2_ESM.pptx]

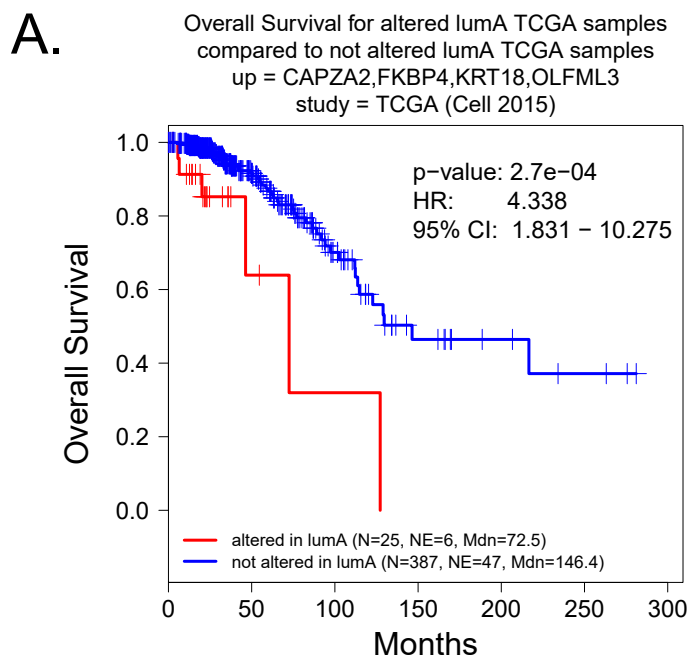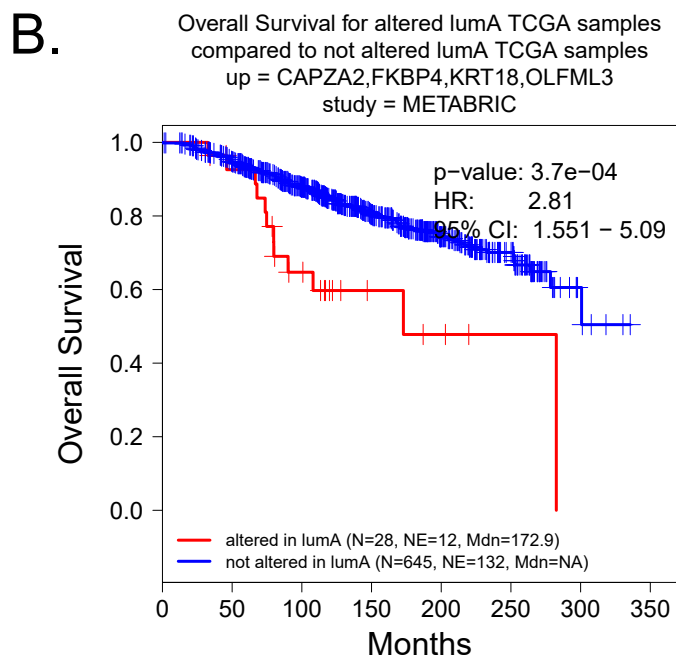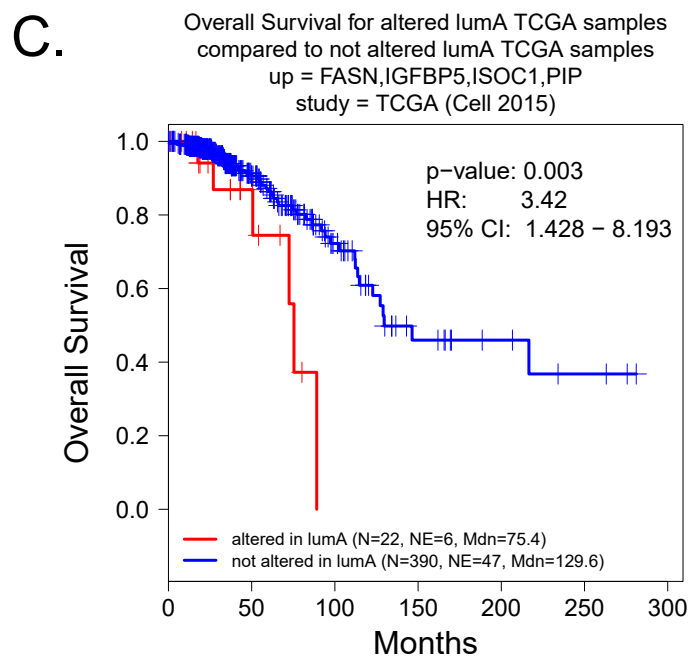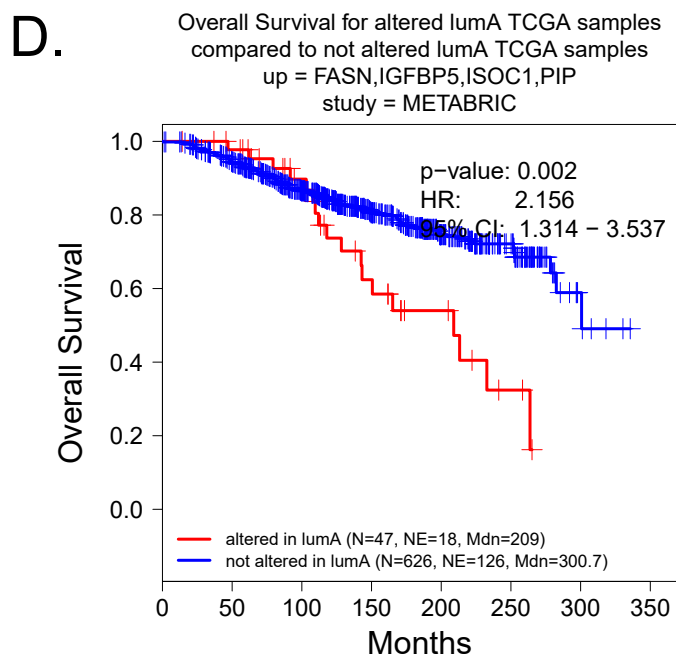

Supplement: Supplementary file 5 — Figure S5. Correlation between Kaplan-Meier survival plots of the clinical outcomes and mRNA co-overexpression of indicated luminal SeCEP genes based on TCGA (left column) and METABRIC (right column) patient data. “N” refers to “Number of patients,” and “NE” refers to “Number of Events (Overall Survival status = DECEASED)”. Each plot shows the log-rank p-value and Hazard Ratio (HR) with 95% Confidence Interval (CI) between the two groups. The red line designates the patient subpopulation showing statistically significant overexpression of the indicated luminal-specific genes (“altered”). The blue line designates the group of patients not showing statistically significant overexpression of the indicated luminal-specific genes (“not altered”). Co-overexpression of distinct sets of genes correlate with statistically significant changes in overall survival in Luminal A patients but not Luminal B or other BC subtypes. (PDF 153 kb) [file 12920_2019_530_MOESM5_ESM.pdf]

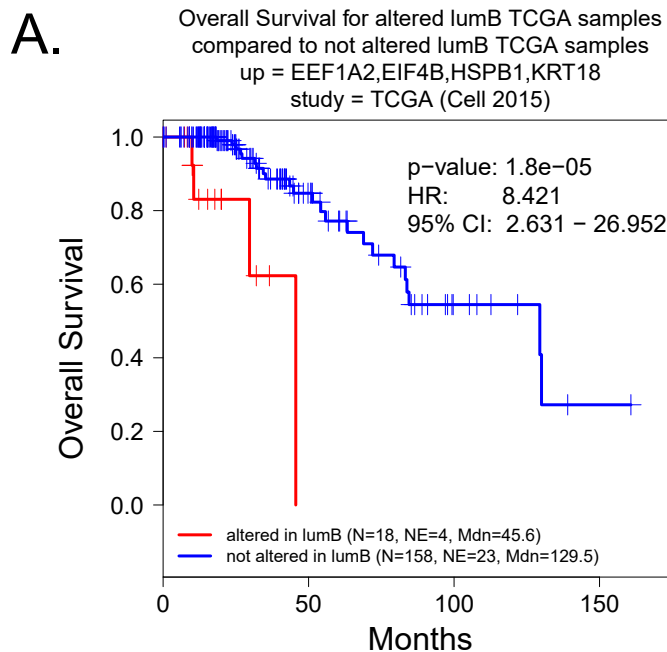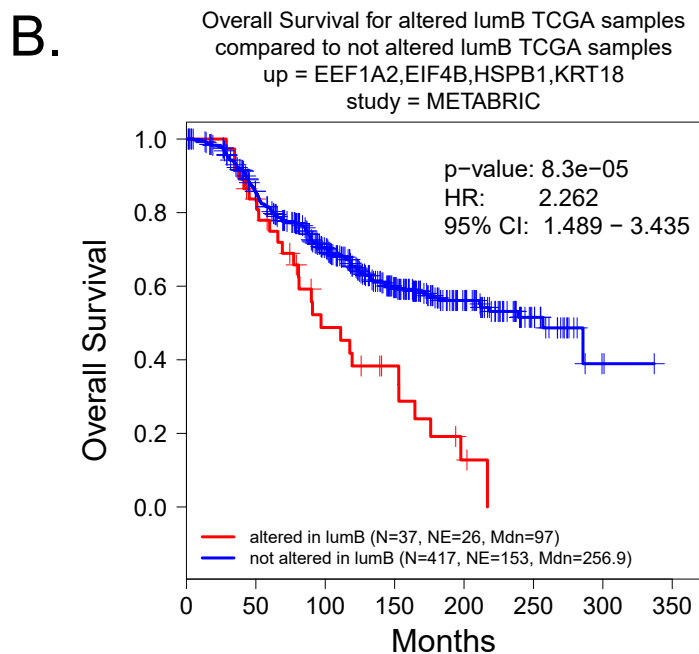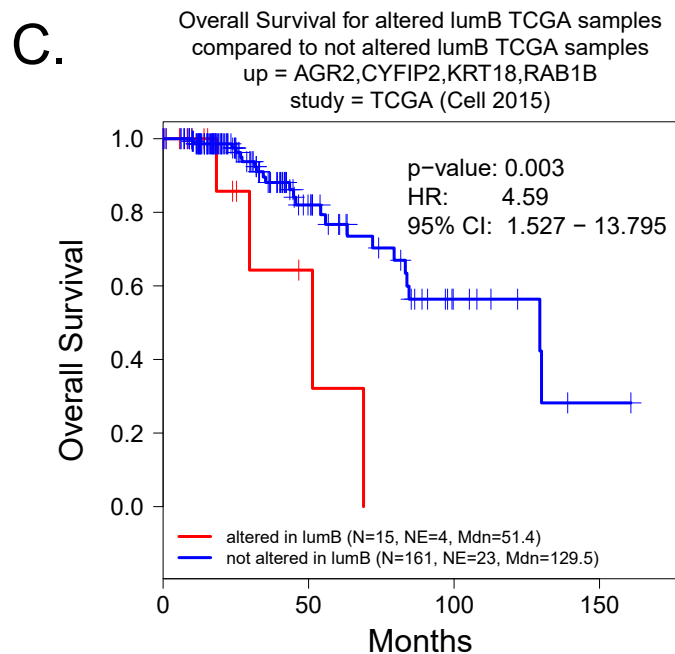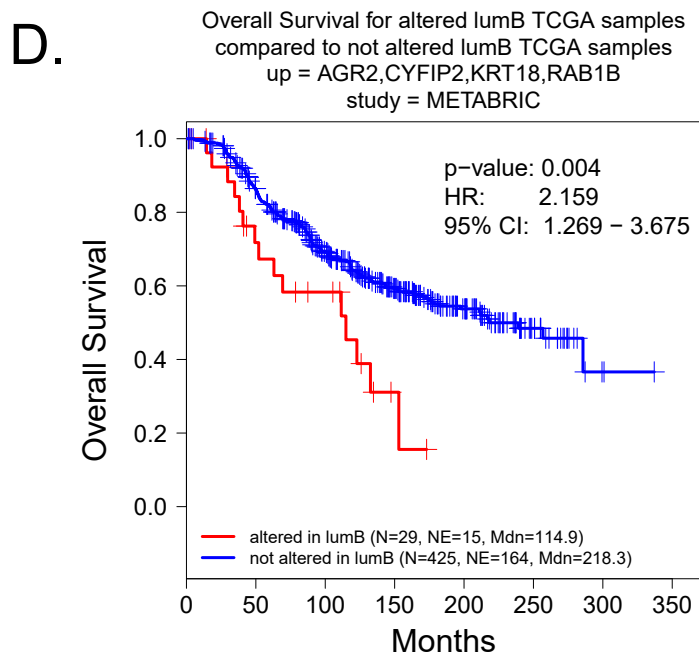

Supplement: Supplementary file 6 — Figure S6. Correlation between Kaplan-Meier survival plots of the clinical outcomes and mRNA co-overexpression of indicated luminal SeCEP genes based on TCGA (left column) and METABRIC (right column) patient data. “N” refers to “Number of patients,” and “NE” refers to “Number of Events (Overall Survival status = DECEASED)”. Each plot shows the log-rank p-value and Hazard Ratio (HR) with 95% Confidence Interval (CI) between the two groups. The red line designates the patient subpopulation showing statistically significant overexpression of the indicated luminal-specific genes (“altered”). The blue line designates the group of patients not showing statistically significant overexpression of the indicated luminal-specific genes (“not altered”). Co-overexpression of distinct sets of genes correlate with statistically significant changes in overall survival in Luminal B patients but not Luminal A or other BC subtypes. (PDF 144 kb) [file 12920_2019_530_MOESM6_ESM.pdf]

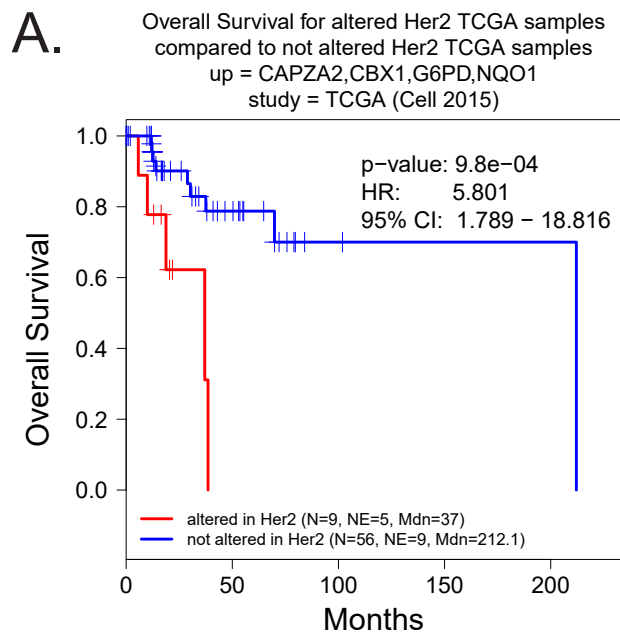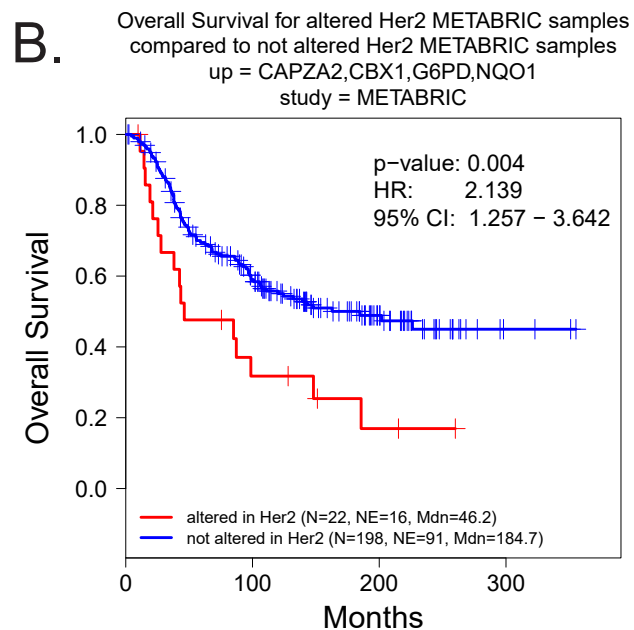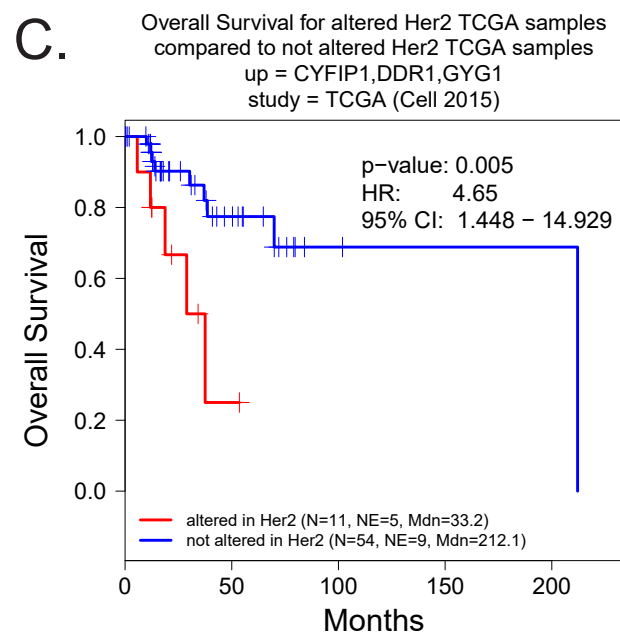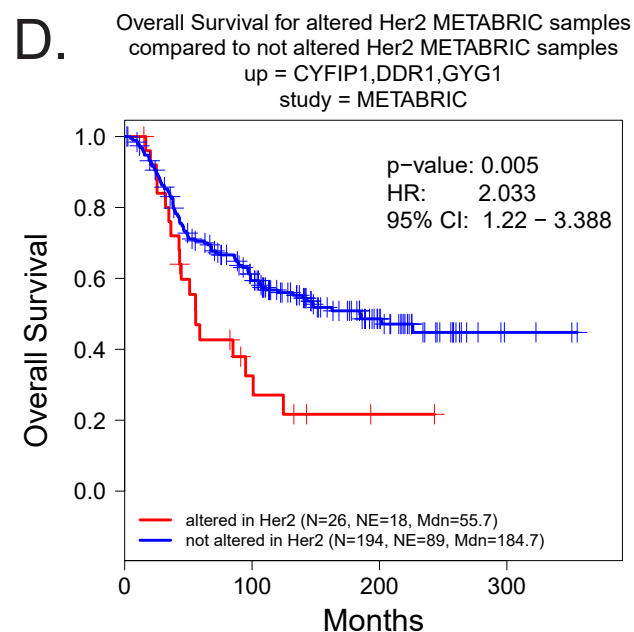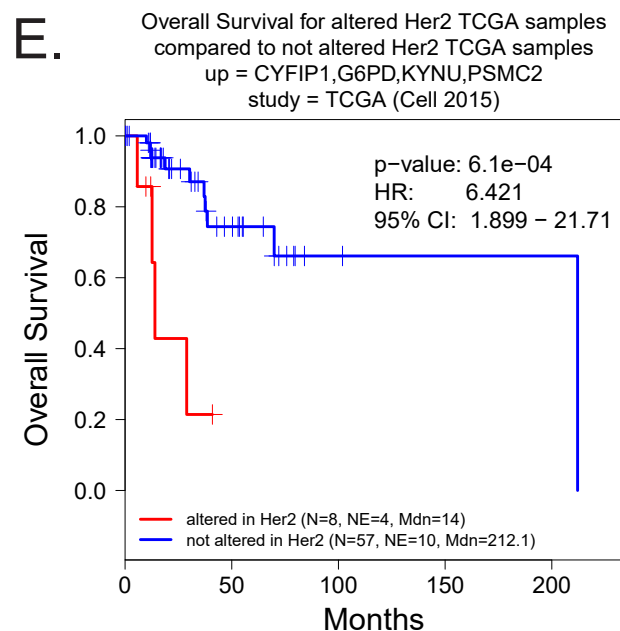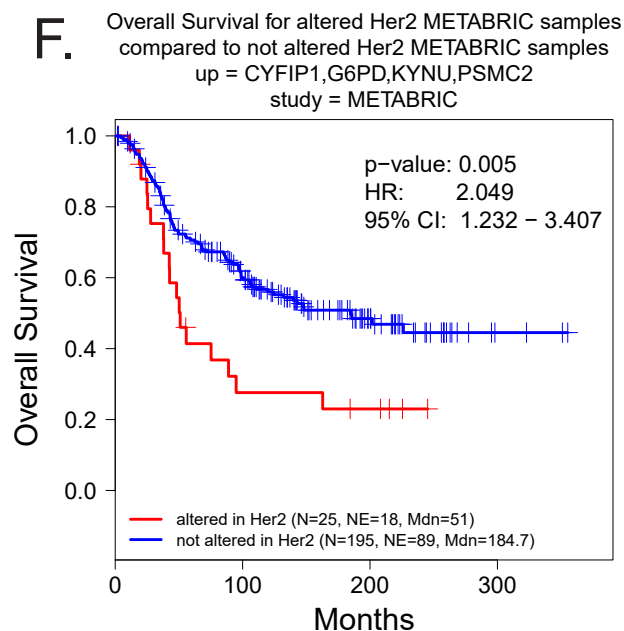

Supplement: Supplementary file 7 — Figure S7. Correlation between Kaplan-Meier survival plots of the clinical outcomes and mRNA co-overexpression of indicated SeCEP genes based on patient data. “N” refers to “Number of patients,” and “NE” refers to “Number of Events (Overall Survival status = DECEASED)”. Each plot shows the log-rank p-value and Hazard Ratio (HR) with 95% Confidence Interval (CI) between the two groups. The red line designates the patient subpopulation showing statistically significant overexpression of the indicated luminal-specific genes (“altered”). The blue line designates the group of patients not showing statistically significant overexpression of the indicated luminal-specific genes (“not altered”). (PDF 792 kb) [file 12920_2019_530_MOESM7_ESM.pdf]
