# Supplementary material for: Novel secretome-to-transcriptome integrated or secreto-transcriptomic approach to reveal liquid biopsy biomarkers for predicting individualized prognosis of breast cancer patients
Source: BMC Med Genomics. 2019 May 30;12:78. doi: 10.1186/s12920-019-0530-7 (PMC6543675; doi:10.1186/s12920-019-0530-7)
Supplement: Supplementary file 3 — Figure S3. A) Over-represented biological processes in the basal-like and luminal BC subtypes. Bars representing the negative log p-values of BLBC process enrichment are displayed in orange and luminal values are in blue. B) Biological functions activated (positive z-score) or suppressed (negative z-score) in the BLBC (orange) and luminal (blue) PAM50 subtypes. C) Pathway activation analysis of PAM50 subtypes. Orange bars represent BLBC values and blue bars represent luminal values. Positive z-scores indicate pathway activation; negative z-scores indicate pathway suppression. (PPTX 53 kb) [file 12920_2019_530_MOESM3_ESM.pptx]

## Slide 1
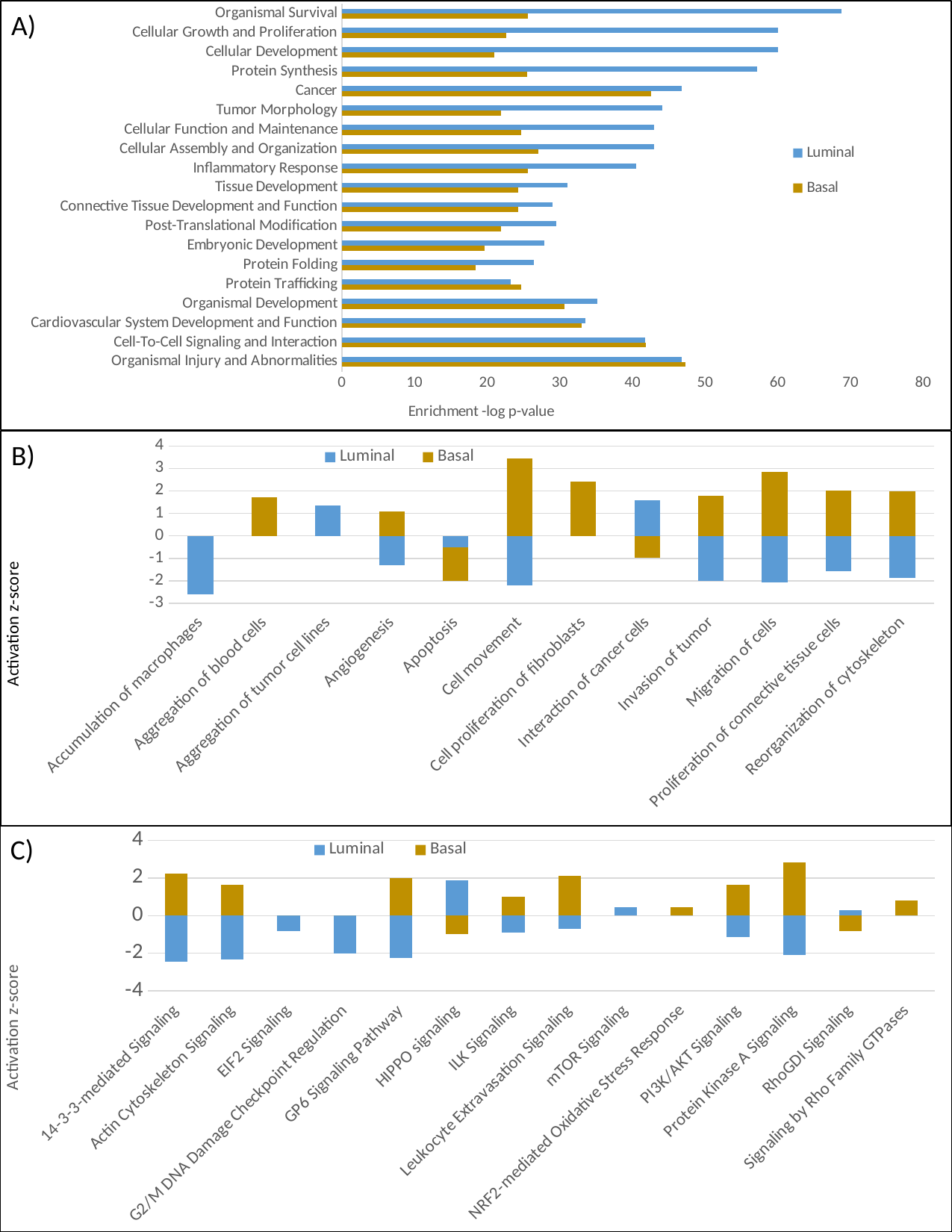

### Chart
| Category | | |
|---|---|---|
| Organismal Injury and Abnormalities | 47.317959504033055 | 46.7262632919467 |
| Cell-To-Cell Signaling and Interaction | 41.790002434018135 | 41.68426318047855 |
| Cardiovascular System Development and Function | 32.96831937534041 | 33.47770610145483 |
| Organismal Development | 30.678261795740063 | 35.12436930184816 |
| Protein Trafficking | 24.69180394281323 | 23.239141371234467 |
| Protein Folding | 18.375752414262536 | 26.37379089792925 |
| Embryonic Development | 19.598144835598983 | 27.890157352582058 |
| Post-Translational Modification | 21.869446857416378 | 29.4859266082598 |
| Connective Tissue Development and Function | 24.250614155678416 | 29.009827583244675 |
| Tissue Development | 24.250614155678416 | 31.049355947431312 |
| Inflammatory Response | 25.641852120837875 | 40.5047408766917 |
| Cellular Assembly and Organization | 27.013732037700592 | 43.00874246089525 |
| Cellular Function and Maintenance | 24.66086023560496 | 43.00874246089525 |
| Tumor Morphology | 21.908668167214095 | 44.09015358651024 |
| Cancer | 42.54351920444819 | 46.7262632919467 |
| Protein Synthesis | 25.546855606902128 | 57.16243749247301 |
| Cellular Development | 20.98745977021622 | 59.98735078591492 |
| Cellular Growth and Proliferation | 22.602732105094635 | 59.98735078591492 |
| Organismal Survival | 25.634318448152467 | 68.79701586865973 |A)
### Chart
| Category | | |
|---|---|---|
| Accumulation of macrophages | -2.588 | None |
| Aggregation of blood cells | None | 1.727 |
| Aggregation of tumor cell lines | 1.359 | None |
| Angiogenesis | -1.302 | 1.082 |
| Apoptosis | -0.526 | -1.482 |
| Cell movement | -2.201 | 3.442 |
| Cell proliferation of fibroblasts | None | 2.415 |
| Interaction of cancer cells | 1.574 | -0.971 |
| Invasion of tumor | -1.994 | 1.795 |
| Migration of cells | -2.075 | 2.852 |
| Proliferation of connective tissue cells | -1.563 | 2.028 |
| Reorganization of cytoskeleton | -1.868 | 1.97 |B)
### Chart
| Category | | |
|---|---|---|
| 14-3-3-mediated Signaling | -2.449 | 2.236 |
| Actin Cytoskeleton Signaling | -2.333 | 1.633 |
| EIF2 Signaling | -0.816 | 0.0 |
| G2/M DNA Damage Checkpoint Regulation | -2.0 | 0.0 |
| GP6 Signaling Pathway | -2.236 | 2.0 |
| HIPPO signaling | 1.89 | -1.0 |
| ILK Signaling | -0.905 | 1.0 |
| Leukocyte Extravasation Signaling | -0.707 | 2.121 |
| mTOR Signaling | 0.447 | 0.0 |
| NRF2-mediated Oxidative Stress Response | 0.0 | 0.447 |
| PI3K/AKT Signaling | -1.134 | 1.633 |
| Protein Kinase A Signaling | -2.111 | 2.828 |
| RhoGDI Signaling | 0.302 | -0.816 |
| Signaling by Rho Family GTPases | 0.0 | 0.816 |C)
