# Supplementary material for: Novel secretome-to-transcriptome integrated or secreto-transcriptomic approach to reveal liquid biopsy biomarkers for predicting individualized prognosis of breast cancer patients
Source: BMC Med Genomics. 2019 May 30;12:78. doi: 10.1186/s12920-019-0530-7 (PMC6543675; doi:10.1186/s12920-019-0530-7)
Supplement: Supplementary file 4 — Figure S4. Protein-protein interaction analysis of A) basal-specific and B) luminal-specific secreted proteins. (PDF 6934 kb) [file 12920_2019_530_MOESM4_ESM.pdf]

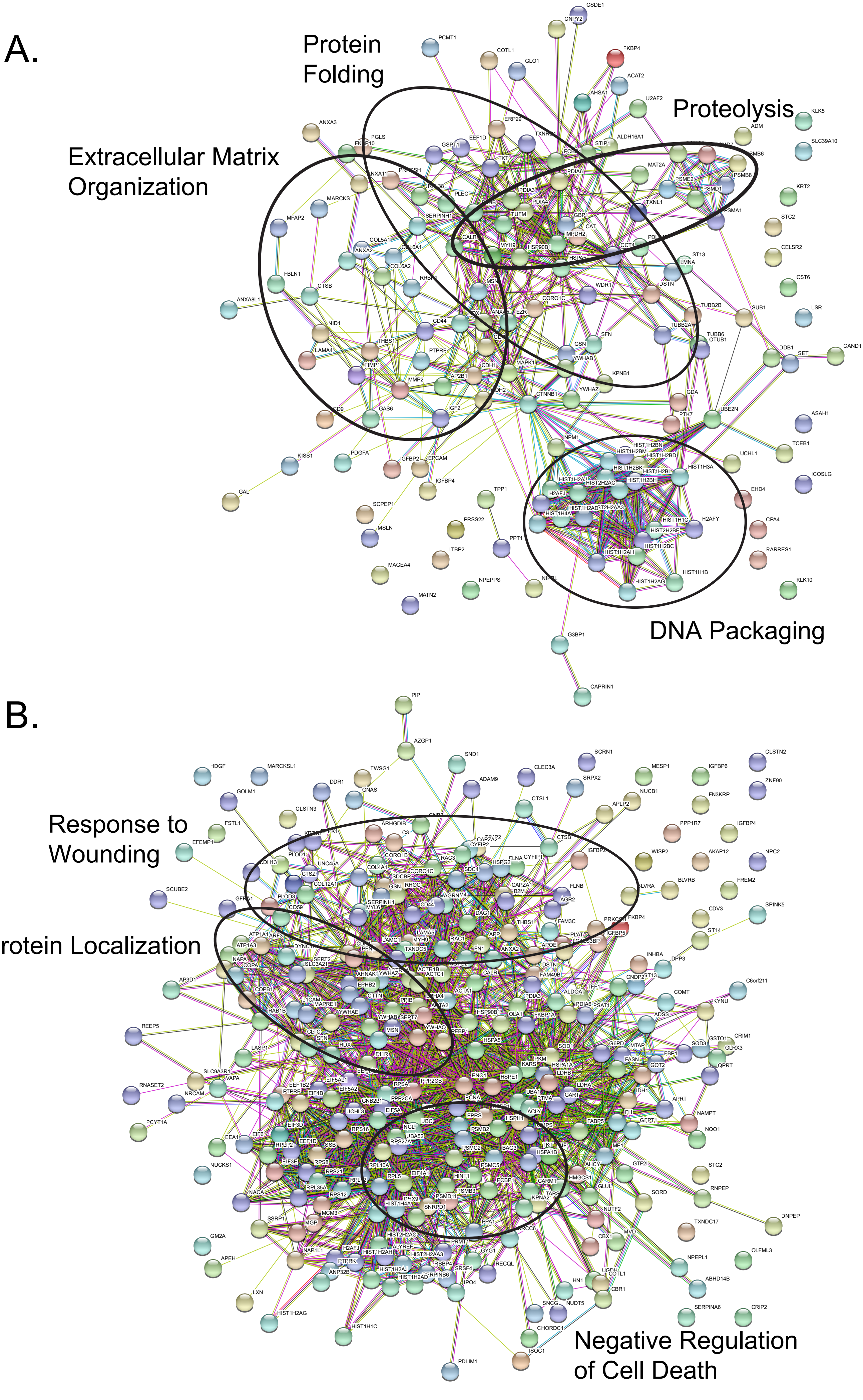

A.
Protein Folding
Proteolysis
Extracellular Matrix Organization
DNA Packaging
B.
Response to Wounding
Protein Localization
Negative Regulation of Cell Death
