# Supplementary material for: Novel secretome-to-transcriptome integrated or secreto-transcriptomic approach to reveal liquid biopsy biomarkers for predicting individualized prognosis of breast cancer patients
Source: BMC Med Genomics. 2019 May 30;12:78. doi: 10.1186/s12920-019-0530-7 (PMC6543675; doi:10.1186/s12920-019-0530-7)
Supplement: Supplementary file 8 — Figure S8. Correlation between Kaplan-Meier survival plots of the clinical outcomes and mRNA co-overexpression of indicated SeCEP genes based on patient data. “N” refers to “Number of patients,” and “NE” refers to “Number of Events (Overall Survival status = DECEASED)”. Each plot shows the log-rank p-value and Hazard Ratio (HR) with 95% Confidence Interval (CI) between the two groups. The red line designates the patient subpopulation showing statistically significant overexpression of the indicated genes (“altered”). The blue line designates the group of patients not showing statistically significant overexpression of the indicated genes (“not altered”). (PDF 121 kb) [file 12920_2019_530_MOESM8_ESM.pdf]

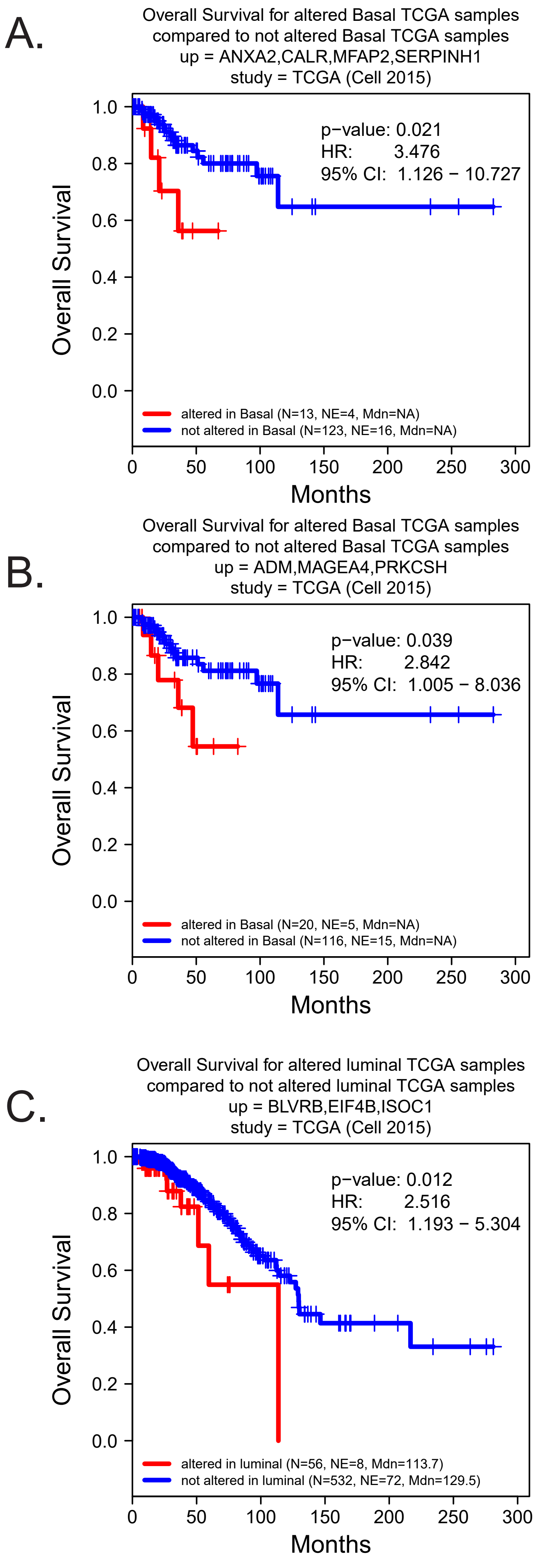
A.
Overall Survival for altered Basal TCGA samples
compared to not altered Basal TCGA samples
up = ANXA2,CALR,MFAP2,SERPINH1
study = TCGA (Cell 2015)
p−value: 0.021
HR: 3.476
95% CI: 1.126 − 10.727
altered in Basal (N=13, NE=4, Mdn=NA)
not altered in Basal (N=123, NE=16, Mdn=NA)
Overall Survival
1.0
0.8
0.6
0.4
0.2
0.0
0
50
100
150
200
250
300
Months
B.
Overall Survival for altered Basal TCGA samples
compared to not altered Basal TCGA samples
up = ADM,MAGEA4,PRKCSH
study = TCGA (Cell 2015)
p−value: 0.039
HR: 2.842
95% CI: 1.005 − 8.036
altered in Basal (N=20, NE=5, Mdn=NA)
not altered in Basal (N=116, NE=15, Mdn=NA)
Overall Survival
1.0
0.8
0.6
0.4
0.2
0.0
0
50
100
150
200
250
300
Months
C.
Overall Survival for altered luminal TCGA samples
compared to not altered luminal TCGA samples
up = BLVRB,EIF4B,ISOC1
study = TCGA (Cell 2015)
p−value: 0.012
HR: 2.516
95% CI: 1.193 − 5.304
altered in luminal (N=56, NE=8, Mdn=113.7)
not altered in luminal (N=532, NE=72, Mdn=129.5)
Overall Survival
1.0
0.8
0.6
0.4
0.2
0.0
0
50
100
150
200
250
300
Months
